# MGAT1 and Complex N-Glycans Regulate ERK Signaling During Spermatogenesis

**DOI:** 10.1038/s41598-018-20465-3

**Published:** 2018-01-31

**Authors:** Barnali Biswas, Frank Batista, Subha Sundaram, Pamela Stanley

**Affiliations:** 1 Department of Cell Biology, Albert Einstein College of Medicine, New York, NY 10461 USA; 2 Present Address: Biochemistry and Molecular Biology Department, University of Debrecen, Debrecen, Hungary

**Keywords:** Cell biology, Developmental biology, Spermatogenesis

## Abstract

Mechanisms that regulate spermatogenesis in mice are important to define as they often apply to fertility in man. We previously showed that conditional deletion of the mouse *Mgat1* gene (*Mgat1* cKO) in spermatogonia causes a germ-cell autonomous defect leading to infertility. MGAT1 is the N-acetylglucosaminyltransferase (GlcNAcT-I) that initiates the synthesis of complex N-glycans. Mechanistic bases of MGAT1 loss were investigated in germ cells from 22- and 23-day males, before any changes in germ cell morphology were apparent. Gene expression changes induced by deletion of *Mgat1* were determined using the Affymetrix gene chip Mouse Mogene 2.0 ST array, and relationships were investigated by bioinformatics including Gene Ontology (GO), Ingenuity Pathway Analysis (IPA), and Gene Set Enrichment Analysis (GSEA). The loss of complex N-glycans promoted the premature up-regulation of genes normally expressed later in spermatogenesis and spermiogenesis, and IPA and GSEA implicated ERK signaling. EGFR and PDGFRA transcripts and ERK1/2 signaling were reduced in 22-day *Mgat1* cKO germ cells. Basigin, a germ cell target of MGAT1, activated ERK1/2 in CHO cells, but not in a Lec1 CHO mutant that lacks MGAT1 and complex N-glycans. Thus, MGAT1 is required to regulate ERK1/2 signaling during spermatogenesis, potentially via different mechanisms.

## Introduction

In mammals, spermatogenesis involves a complicated sequence of cell-cell interactions and signaling pathways^1,2^. In order to identify roles for glycans in spermatogenesis, we previously generated a number of conditional mutants of protein glycosylation by deleting various glycosyltransferase genes in spermatogonia at 3 days post-partum (dpp) using a Stra8-iCre transgene^3^. Deletion of *C1galt1* that generates core 1 and 2 O-glycans, or deletion of *Pofut1* that transfers O-fucose to Notch receptors and is required for Notch signaling, had no major effects on spermatogenesis, but deletion of *Mgat1* blocked spermatogenesis. *Mgat1* conditional mutant (cKO) males exhibit multinuclear cells (MNC) and produce no sperm^3^. The *Mgat1* gene encodes N-acetylglucosaminyltransferase I (GlcNAcT-I), the transferase that transfers GlcNAc from UDP-GlcNAc to Man_5_GlcNAc_2_Asn to generate hybrid and complex N-glycans^4,5^. In the absence of MGAT1, N-glycans of mature glycoproteins are solely oligomannosyl, and lack all branch antennae that contain GlcNAc, Gal, Fuc, and sialic acid^6^. Global inactivation of the mouse *Mgat1* gene leads to embryonic lethality at approximately E9.5^7,8^.

The architecture of seminiferous tubules in sections from 7 week *Mgat1* cKO mice is disrupted^3^. All tubules contain MNC or symplasts composed of fused spermatids, and lack sperm. A related phenotype is observed with the inactivation of the alpha-mannosidase IIx gene *Man2a2*, that encodes a mannosidase that acts immediately after MGAT1 in the generation of complex N-glycans. *Man2a2* null mice are infertile and also exhibit MNC in testis tubules^9^. Interestingly, loss of the glycoprotein basigin, a carrier of complex N-glycans in germ cells generated by MGAT1^3^, also gives rise to MNC and infertility^10^. In this paper, we determine the earliest time when loss of MGAT1 causes a change in germ cell organization. We show that, at a stage when Sertoli cells, spermatogonia and spermatocyte numbers are not affected in 22 and 23 dpp *Mgat1* cKO testes, molecular changes have nevertheless occurred that lead to the premature expression of spermiogenic genes, and to reduced ERK1/2 signaling. In addition, we show that basigin, a target of MGAT1 in germ cells^3^, does not stimulate pERK1/2 levels in Lec1 CHO cells expressing only oligomannosyl N-glycans (a model for *Mgat1* cKO germ cells). In contrast, basigin with complex N-glycans stimulates ERK1/2 signaling in wild type CHO cells.

## Results

### Early testicular changes associated with deletion of *Mgat1* in spermatogonia

**Figure 1:**
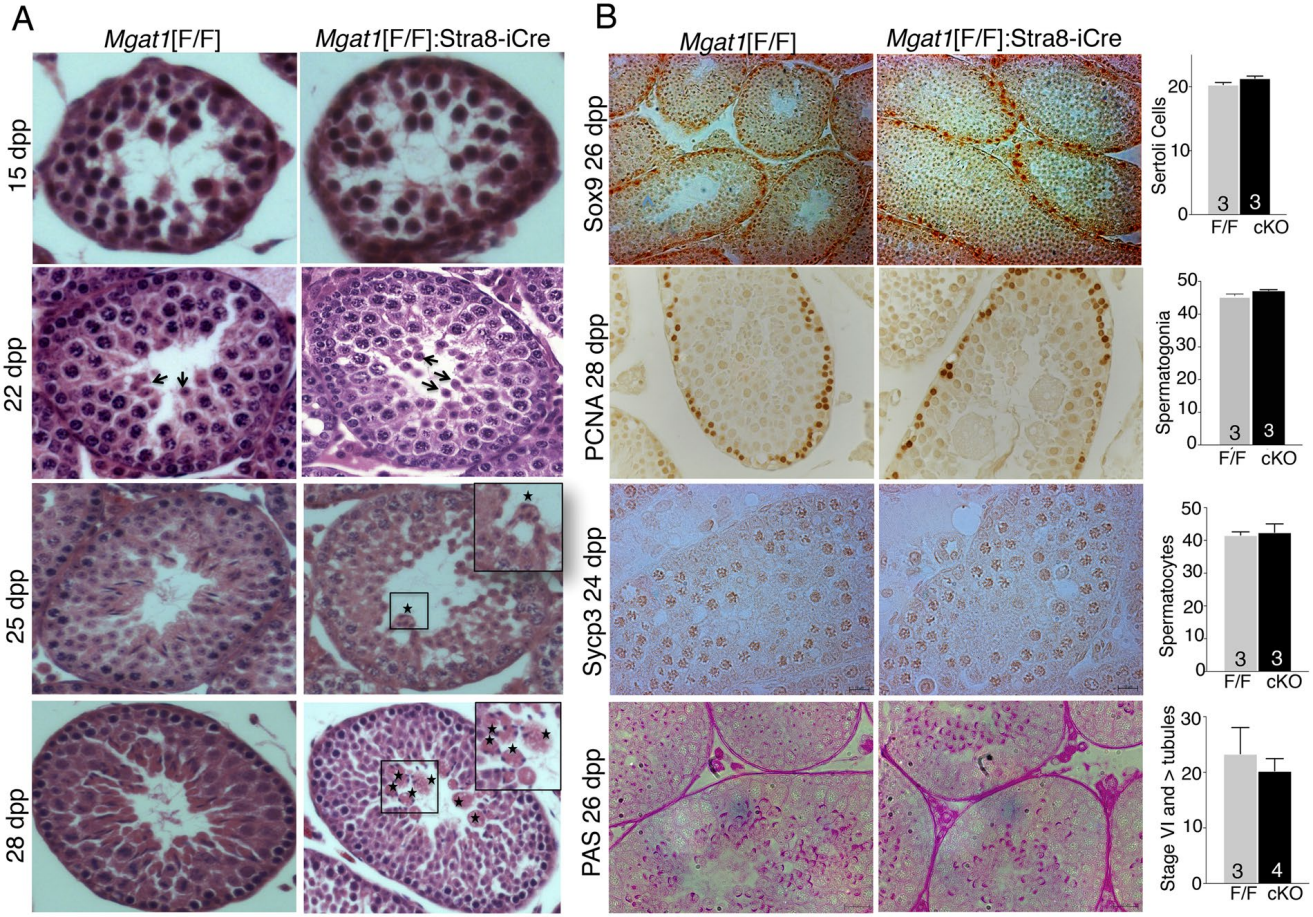
Onset of morphological changes in *Mgat1*[F/F]:Stra8-iCre testes. (**A**) Representative control *Mgat1*[F/F] and *Mgat1*[F/F]:Stra8-iCre testis sections stained with hematoxylin and eosin at 15–28 dpp (n = 3–4 mice per genotype; 20–30 tubules observed per section). At 22 dpp round spermatids were present in control and *Mgat1* cKO tubules (arrows). At 25 dpp, few elongated spermatids were seen in *Mgat1* cKO tubules and some MNC were observed in a few tubules (asterisks, inset). At 28 dpp, *Mgat1* cKO tubules contained MNC comprised of fused spermatids (asterisks, inset and Supplementary Fig. S1). Images were scanned at 40× in a Perkin Elmer Scanner. (**B**) Sertoli cells were identified by anti-Sox9 Ab, spermatogonia by anti-PCNA Ab; primary spermatocytes by anti-Sycp3, and acrosomes by PAS. Histograms show numbers of stained cells per 20–30 tubules per section, and numbers of mice examined. Tubules at stage VI and beyond based on morphology with PAS+ acrosomes were counted per 50 tubules. Histograms represent mean ± SEM. Images were photographed at 20×. Controls for antibody specificity are shown in Supplementary Fig. S2.

Our previous study characterized *Mgat1*[F/F]:Stra8-iCre males at 7 weeks^3^. To identify the earliest stage at which defective spermatogenesis is observed, testes from control and *Mgat1* cKO males from 15 to 28 dpp were compared by histology (Fig. 1A). At 15 dpp, no apparent differences in seminiferous tubule size or the population of germ cells present in 50 tubules were observed (n = 3 mice/group). At 22 and 23 dpp, round spermatids were present in both control and mutant tubules, and there were still no apparent histological differences (Fig. 1A). At 24 and 25 dpp, fusion of cells adjacent to the lumen was observed in a few tubules (Supplementary Table S1; Fig. 1A). Spermatids were identified based on nuclear size, morphology, location in the tubule or detection of acrosomes by periodic Schiff stain (PAS) at 22–25 dpp (Fig. 1A,B), or the acrosomal protein sp56 at 28 dpp (Supplementary Fig. S1). At 28 dpp, mature spermatozoa were present in control but not *Mgat1* cKO mutant testis sections (Fig. 1A). The number of tubules with elongated spermatids was significantly reduced in 28 dpp mutant testes, and MNC were present (Supplementary Table S1). *Mgat1* cKO and control testis sections were analyzed at 24–26 dpp to detect Sertoli cells (SOX9), spermatogonia (PCNA), spermatocytes (SYCP3), and spermatids (PAS) (Fig. 1B; Supplementary Fig. S2). The number of Sertoli cells, spermatogonia, spermatocytes and Stage VI and beyond tubules were not significantly reduced in *Mgat1* cKO versus control tubules (Fig. 1B histograms).

### Gene expression in *Mgat1* cKO testicular germ cells

To gain insights into molecular mechanisms that occur early, before morphological changes are apparent, and thus give rise to the defective spermatogenesis of *Mgat1* cKO males, microarray analyses were performed on RNA from germ cells from 22 and 23 dpp males (Supplementary Table S2). To investigate the relative purity of germ cells, qRT-PCR for cell-type specific genes was performed on cDNA prepared from 22 dpp germ cells and compared to 22 dpp testis. Transcripts of *Rhox5* (Sertoli cells) and *Cyp11a1* (Leydig cells) were greatly reduced in germ cell compared to testis preparations (Supplementary Fig. S3A). By contrast, the expression of germ cell-specific genes *Sycp3* (spermatocytes) and *Dbil5* (round spermatids) were similar in testis and germ cell preparations. *Acrv* transcripts (late spermatids) were poorly represented in germ cell preparations. Importantly, the expression of *Mgat1* was greatly reduced in *Mgat1* cKO germ cells at 22 and 23 dpp (Supplementary Fig. S3B). PCR genotyping of germ cell genomic DNA^11^ showed that *Mgat1* cKO germ cells contained only deleted *Mgat1* alleles (520 bp), whereas control germ cells contained only floxed *Mgat1* alleles (560 bp; Supplementary Fig. S3C). Therefore, *Mgat1* transcripts in germ cell preparations were contributed by a small fraction of non-germ cells.

**Figure 2:**
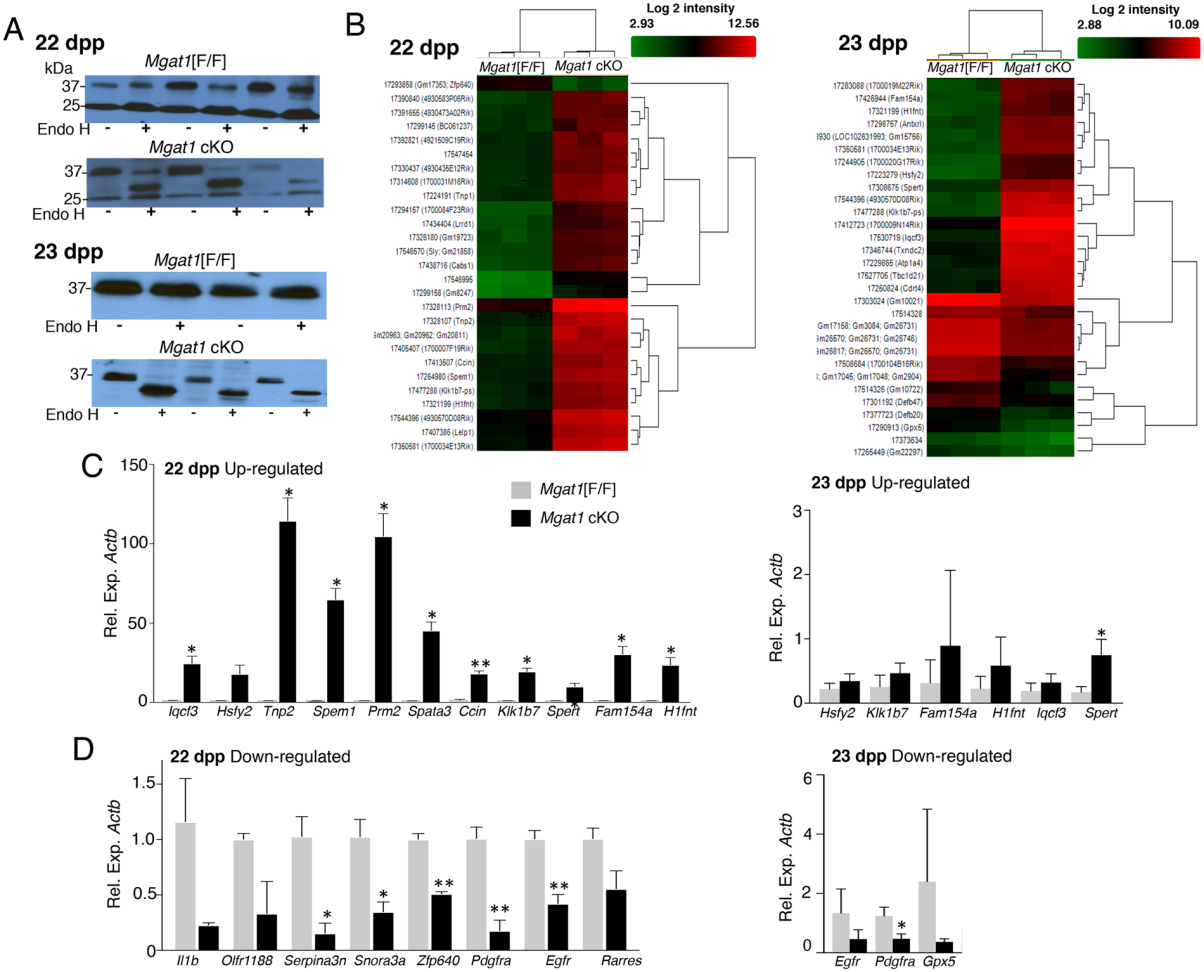
Gene expression changes in *Mgat1* cKO germ cells. (**A**) Western blot analysis for basigin before and after endoglycosidase H treatment of germ cell lysates. Basigin from *Mgat1* cKO germ cells was sensitive to Endo H digestion, consistent with a lack of complex N-glycans. (**B**) Hierarchical clustering of genes expressed in control versus *Mgat1* cKO germ cell cDNA preparations from 22 dpp males, and from 3 replicate cDNA preparations of control versus *Mgat1* cKO germ cell RNA pools obtained from 6 mice per genotype at 23 dpp. Red indicates high and green indicates low relative expression. (**C**) qRT-PCR validation of up-regulated genes in microarray data using cDNA from individual preparations used in microarray experiments. Bars represent mean ± SEM (gray, control; black *Mgat1* cKO). Data are from two experiments performed in duplicate (n = 3 mice/group). *p < 0.05, **p < 0.01. (**D**) qRT-PCR of down-regulated genes identified in microarray data performed on the same RNA samples used in B. Data are from two experiments performed in duplicate (n = 3 mice/group). *p < 0.05, **p < 0.01. Both control and *Mgat1* cKO transcripts were determined relative to *Actb*.

The nature of the N-glycans on basigin (~37 kDa), a germ cell glycoprotein target of MGAT1^3^, confirmed efficient deletion of *Mgat1* at 22 and 23 dpp. Thus, control germ cell basigin with complex N-glycans was resistant to digestion by endoglycosidase H (Endo H). By contrast, *Mgat1* cKO germ cell basigin was sensitive to Endo H digestion, showing that it carried oligomannosyl N-glycans (Fig. 2A and Supplementary Fig. S3D). Incomplete Endo H digestion probably accounts for traces of undigested basigin in some *Mgat1* cKO lysates. Importantly, comparison with ~25 kDa non-glycosylated basigin observed at 22 dpp, showed that levels of basigin were similar in control and *Mgat1* cKO germ cells (Fig. 2A).

### Differentially expressed genes (DEGs) in control versus *Mgat1* cKO germ cells

Gene expression profiles of 22 and 23 dpp testicular germ cells from control and *Mgat1* cKO males were determined using the GeneChip™ Mouse Gene 2.0 ST Array (34,472 genes). For the 22 dpp experiment, cDNA from three mice per group was analyzed (Supplementary Table S2). For the 23 dpp experiment, RNA (RIN > 9) aliquots from six control and six *Mgat1* cKO germ cell preparations were separately pooled, and three aliquots from the control and *Mgat1* cKO pool, respectively, were converted to cDNA for analysis. Affymetrix Expression Console was used to process the .CEL files of the array. .CHP files were generated using the RMA sketch workflow after signal summarization and data normalization. Gene level analysis was further conducted with Affymetrix Transcriptome Analysis Console v2.0 software (TAC). Hierarchical clustering differentiated control and *Mgat1* cKO samples (Fig. 2B). Volcano plots representing gene distributions are shown in Supplementary Fig. S4. Genes that were differentially expressed based on ANOVA *p* < 0.05 and FDR *p* < 0.05 were identified. There were 1,643 DEGs in 22 dpp *Mgat1* cKO versus control germ cells, with an absolute fold-change (linear) of <−2 to >+2.0. Of those, 1,400 genes were up-regulated and 243 down-regulated at 22 dpp. At 23 dpp, 784 genes were differentially regulated at <−2 to >+2.0, 771 genes up-regulated, and 13 genes down-regulated in *Mgat1* cKO germ cells. Many of the DEGs in *Mgat1* cKO germ cells (Supplementary Table S3) are involved in spermatogenesis and later stages of spermiogenesis^12^. Validation of microarray experiments by qRT-PCR was performed on cDNA prepared from germ cell RNA obtained from the individual mice used in microarray experiments (Fig. 2C,D). PCR primers are given in Supplementary Table S4. Microarray data are deposited in NCBI’s Gene Expression Omnibus (GEO) and are accessible through GEO serial accession number GSE99035.

### Enriched Gene Ontology (GO) analysis

DEGs identified by microarray were subjected to GO analysis, which classified 513 DEGs into three GO categories. The most enriched GO terms that correlated with *Mgat1* cKO DEGs were sperm egg recognition (GO:0035036, Biological Process, enrichment score (ES) 9.41, Bonferroni count (BC) 8,371), lysozyme activity (GO:0003796, Molecular Function, ES 24.17, BC 3,018), and sperm fibrous sheath (GO:0035686, Cellular Component (CC), ES 21.76, BC 1,288). The number of genes present in the 22 dpp dataset for each GO function are shown in Supplementary Fig. S5, with fold-enrichment of input genes over expected.

### Enriched biological pathways by Ingenuity Pathway Analysis (IPA)

**Figure 3:**
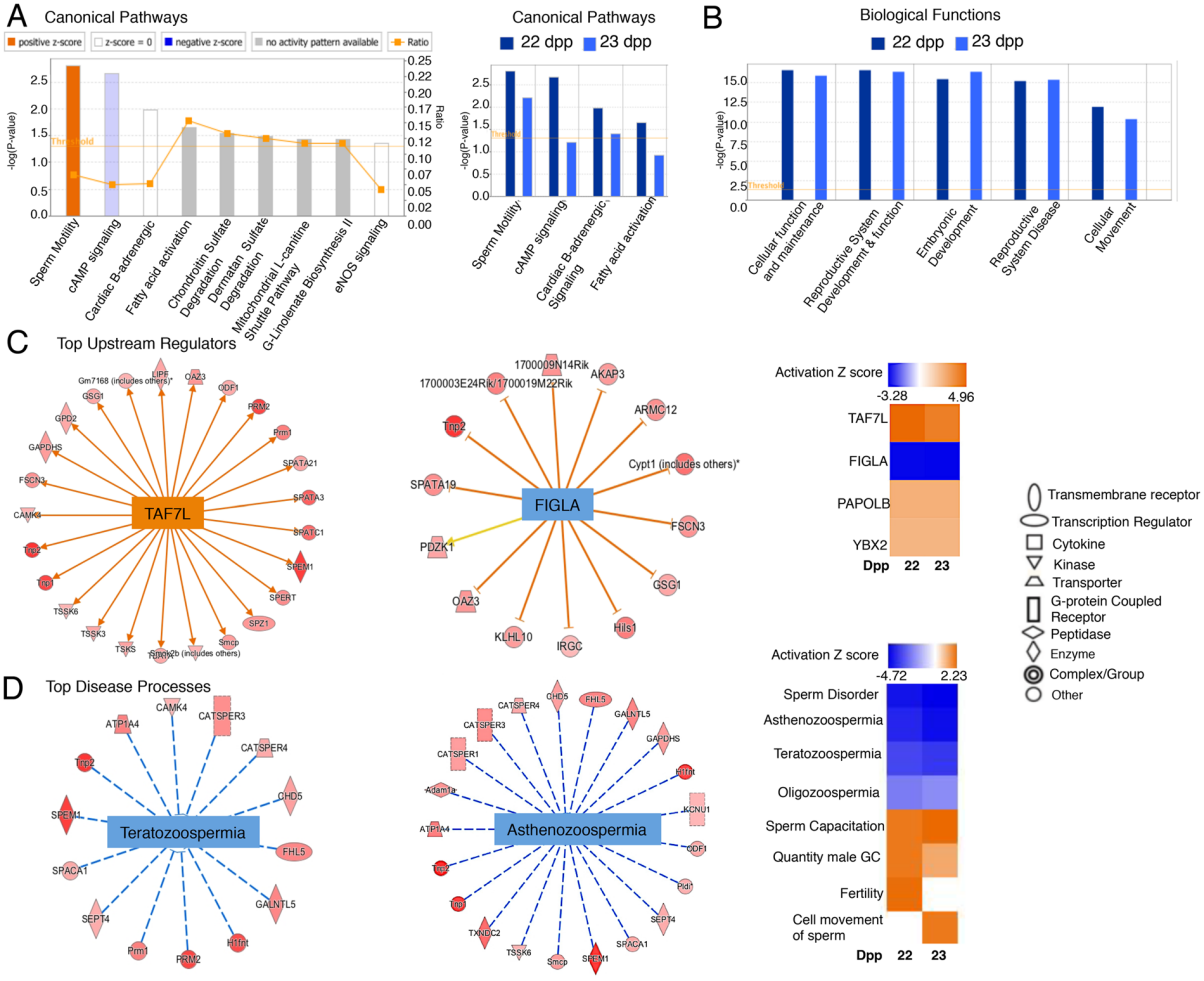
IPA of DEGs in control versus *Mgat1* cKO germ cells. (**A**) Canonical pathways significantly overrepresented in *Mgat1* cKO germ cells compared to control according to their -log *p* value. Colors indicate the activation Z score of processes: activated processes are orange, while inhibited processes are blue. No activity pattern is represented by gray, and no change is denoted by white. Right panel shows comparison analysis of canonical pathways from independent 22 dpp versus 23 dpp mutant germ cells based on *p* value. (**B**) Comparison of biological functions based on *p* value in 22 versus 23 dpp shows Cellular Function and Maintenance and Reproductive System Development and Function as the top hits in both 22 and 23 dpp DEGs. (**C**) IPA upstream regulators of DEGs in control versus *Mgat1* cKO 22 dpp germ cells. Symbols of target proteins or upstream regulators in red indicate a predicted increase or activation. The symbol shapes denote the molecular classes of proteins. Right panel shows the heat map for comparison of upstream regulators at 22 and 23 dpp based on activation Z score. (**D**) IPA downstream effect analysis networks associated with diseases and functions. Z scores for both diseases are negative indicating a reduction in disease. Edges and nodes are color-coded based on the predicted relationship as indicated in the legend. Right panel shows the heat map for comparison of disease conditions at 22 and 23 dpp based on activation Z score.

Using IPA, relationships between significant DEGs of absolute fold-change ±1.5 with adjusted FDR and ANOVA *p* < 0.05 were examined to determine the most significant canonical pathways and biological networks altered in *Mgat1* cKO germ cells. In 22 dpp germ cells (1,086 genes), the top canonical pathway was connected with sperm motility (Fig. 3A). A comparison of canonical pathways affected in 22 versus 23 dpp germ cells shows them to be similar (Fig. 3A, right panel). The significance value is a measure of the likelihood that genes from the input dataset participate in a pathway or function. Sperm motility was ranked third in the 23 dpp dataset. A comparison of biological functions regulated by *Mgat1* cKO at 22 versus 23 dpp is shown in Fig. 3B. The top biological functions in both 22 and 23 dpp *Mgat1* cKO germ cells were Cellular Function and Maintenance, Reproductive System Development, and Function and Embryonic Development (Fig. 3B).

### Top upstream transcriptional regulators

IPA upstream functional analysis was used to predict the top upstream transcriptional regulators in DEGs of *Mgat1* cKO germ cells at 22 and 23 dpp based on their gene targets. An overlap *p* value was calculated on the basis of significant overlap between genes in the test dataset and target genes regulated by the same regulator in the IPA knowledge base. The activation Z score algorithm was used to make predictions. IPA predicted the top transcriptional regulator activated in the 22 and 23 dpp datasets to be TAF7L (Z score 4.96; overlap *p* value 8.89E-31 for 22 dpp; Z score 4.24, overlap *p* value 6.16E-24 for 23 dpp; Fig. 3C). FIGLA is predicted to be inhibited in both the 22 dpp (Z score −3.22; overlap *p* value 1.56E-12) and 23 dpp datasets (Z score −3.28, *p* 1.17E-11) (Fig. 3C). Target genes of TAF7L were up-regulated in our datasets, and predict activation of this regulator. For FIGLA, the target genes from the input datasets were up-regulated, but the regulator is predicted to inhibit transcription. Comparison analysis for upstream regulators at 22 and 23 dpp showed similar activation Z scores for each of the top regulators at both developmental stages (Fig. 3C, right panel).

**Figure 4:**
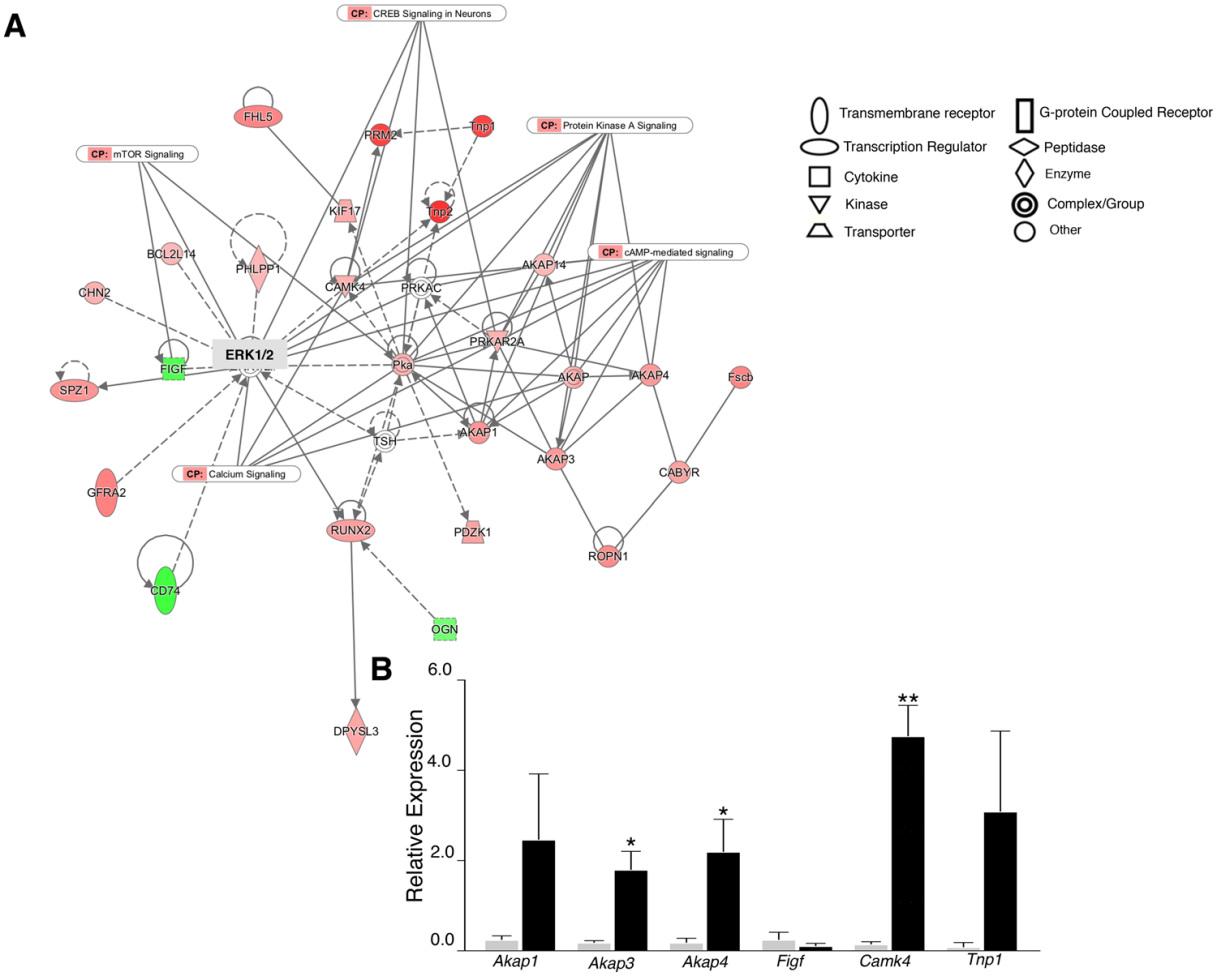
The top network identified by IPA in 22 dpp *Mgat1* cKO germ cells. (**A**) Molecules are represented as nodes (see legend). Nodes in red or green represent up-regulated or down-regulated genes, respectively. (**B**) Validation of Network 1 by qRT-PCR performed in duplicate on germ cell cDNA prepared from 3 mice in each group. Gray bars represent control and black bars represent *Mgat1* cKO germ cells (mean ± SEM; *p < 0.05, **p < 0.01).

We tested whether the expression of TAF7L itself was up-regulated by IHC, qRT-PCR and western blot analyses (Supplementary Fig. S6). TAF7L is expressed in the nucleus or cytoplasm of germ cells, depending on their stage of differentiation^13^. We observed TAF7L in the cytoplasm of spermatocytes at 23 dpp. There was no apparent difference in the intensity of the signal between control and *Mgat1* cKO sections (Supplementary Fig. S6A). By qRT-PCR, TAF7L transcripts in 23 dpp *Mgat1* cKO germ cells were slightly reduced (Supplementary Fig. S6B). However, TAF7L protein levels were similar by western blot analyses of germ cell lysates (Supplementary Fig. S6C). Nevertheless, microarray data showed up-regulation of numerous TAF7L target genes (Fig. 3C), and qRT-PCR data validated several of these genes including *Spert*, *Spem1*, *Prm2*, *Spata3*, *Tnp1*, and *Tnp2* (Figs 2C, 4B). Thus, TAF7L activation leading to up-regulation of target genes appears to be due to factors other than increased expression of TAF7L.

### Correlation with disease genes

DEGs of *Mgat1* cKO germ cells were compared by IPA with genes implicated in diseases. Disease categories impacted in 22 and 23 dpp *Mgat1* cKO germ cells include Teratozoospermia and Asthenozoospermia diseases of men. Up-regulated DEGs from the 22 dpp dataset are shown in Fig. 3D. The blue dotted lines indicate the predicted suppression of disease in *Mgat1* cKO germ cells, as up-regulation of spermatogenic and spermiogenic genes is predicted to reduce development of diseases such as Teratozoospermia and Asthenozoospermia. Comparison of heat maps for disease conditions at 22 and 23 dpp shows that sperm disorder, Asthenozoopermia, Teratozoospermia, and Oligoszoospermia, as defined in men with fertility disorders, were reduced, whereas sperm capacitation and quantity of male germ cells were increased (Fig. 3D, right panel). Genes related to fertility were increased relatively more at 22 than 23 dpp, whereas genes related to cell movement of sperm were relatively increased at 23 compared to 22 dpp (Fig. 3D).

### Interaction networks of DEGs

IPA was used to map biological relationships of the *Mgat1* cKO germ cell DEGs into networks based on published literature. At 22 dpp, a 26-gene network with a score of 44 was the most significant. The functions associated with network 1 are cellular development, reproductive system development and function, and cell morphology. The central molecule in this network is ERK1/2, but it is not indicated as activated or inhibited, and neither ERK1 nor ERK2 are amongst the DEGs in our datasets. PKA, several AKAPs and CAM kinase 4 genes were predicted to be up-regulated. Spermiogenesis-specific genes that include transition protein 2 (*Tnp2*) and protamine 2 (*Prm2*) were also predicted to be up-regulated (Fig. 4A). Some extracellular matrix genes such as laminin and collagen, indirectly associated with network 1 and not predicted to change, were removed for clarity. The genes in network 1 overlapped with canonical pathways that show a direct or indirect relation with ERK1/2 (Fig. 4A). Five of the up-regulated genes (*Akap1*, *Akap3*, *Akap4*, *Tnp1*, *Camk4*) and 1 down-regulated gene (*Figf*) were validated by qRT-PCR (Fig. 4B). *Camk4* is a protein kinase that phosphorylates protamines^14^. Network 1 from the 23 dpp dataset is associated with activation of TAF7L and YBX2 target genes (not shown). This network had a score of 28 with 16 associated molecules, including genes contributing to cellular development, cellular growth, proliferation and embryonic development. The central molecule in network 1 is TAF7L, and PAPOLB, FIGLA and YBX2 all contribute to the up-regulation of DEGs in the 23 dpp dataset. Additionally, a group of genes involved in metabolism are significantly up-regulated upon loss of *Mgat1*, and likely reflect both metabolic and spermatogenic functions.

### Gene set enrichment analysis (GSEA)

**Figure 5:**
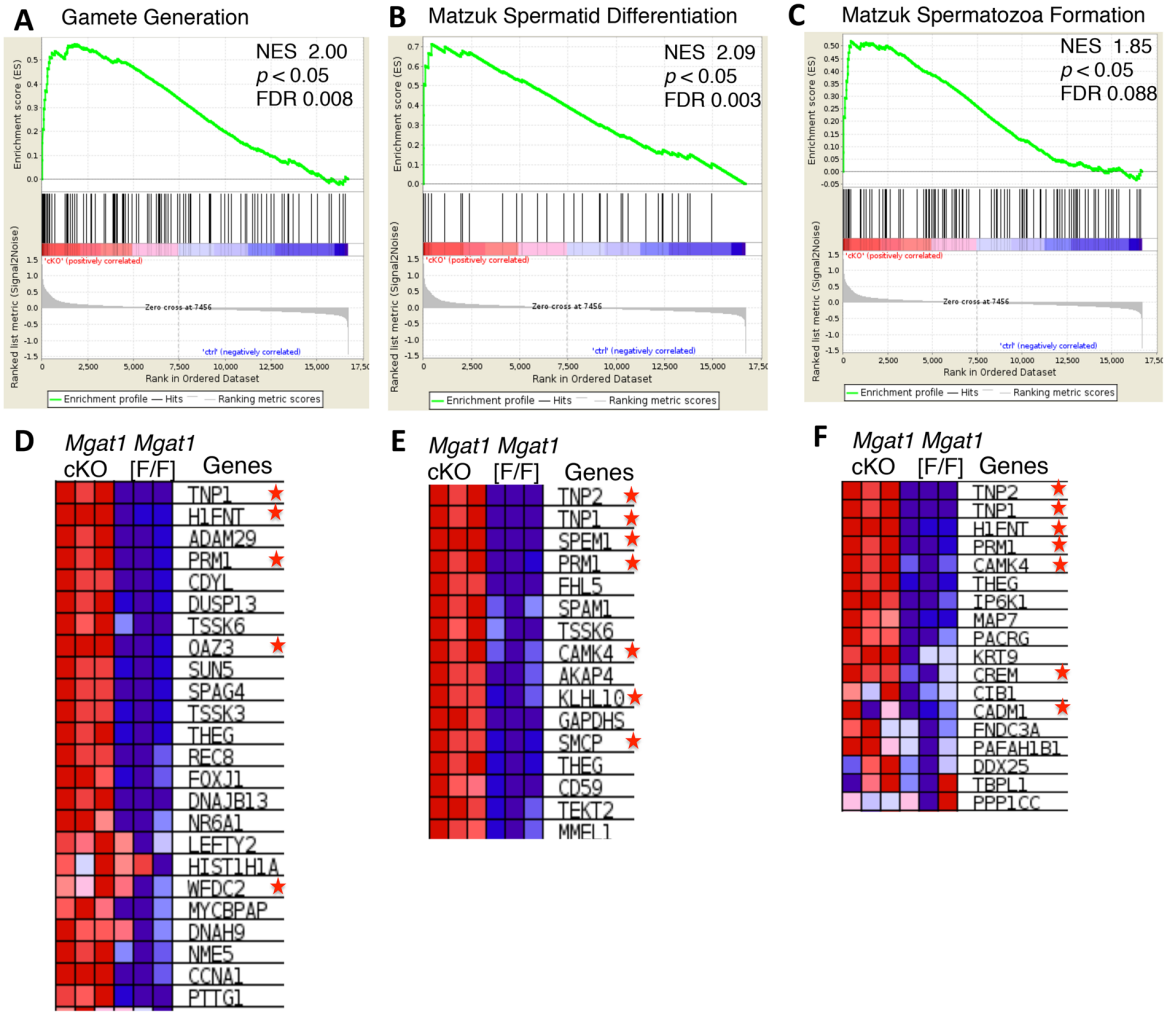
Gene set enrichment analysis (GSEA) of microarray data in 22 dpp* Mgat1* cKO germ cells. GSEA enrichment plots of three gene clusters that are enriched in *Mgat1* cKO germ cells were (**A**) Gamete Generation, (**B**) Matzuk Spermatid Differentiation, and (**C**) Matzuk Spermatozoa Formation. Heat map of positively-enriched genes in *Mgat1* cKO germ cells compared to control for (**D**) gamete generation (**E**) Matzuk spermatid differentiation and (**F**) Matzuk spermatozoa formation. *Genes involved in spermiogenesis that are up-regulated prematurely in *Mgat1* cKO males at 22 dpp.

We utilized GSEA to evaluate *Mgat1* cKO DEGs at the level of published, classified gene sets in MSigDB. At 22 dpp, 15 of the 3,658 gene sets in the C2 (curated) gene sets were significantly enriched in *Mgat1* cKO, and 1975 were significantly enriched in control germ cells (FDR < 0.25). The most significant C2 gene sets were Matzuk spermatid differentiation and Matzuk spermatozoa formation, and the top C5 (GO) gene set was gamete generation (Fig. 5). Genes involved in spermiogenesis, or expressed in the later stages of spermatogenesis (*Tnp1*, *Tnp2*, *Prm1*, *Crem*, *Cadm1*, *Ybx2*), are among the top up-regulated genes (Fig. 5D‒F; stars). GSEA of 23 dpp *Mgat1* cKO germ cell DEGs identified 6 of the C2 gene sets were significantly enriched (FDR < 0.25) in *Mgat1* cKO germ cells. Top C2 gene sets were Matzuk spermatozoa (NES 2.14) and Matzuk spermatid differentiation (NES 1.99), and top C5 gene set was gamete generation (NES 2.0), similar to 22 dpp germ cells. Thus, germ cells lacking *Mgat1* exhibit premature expression of genes that promote spermatogenesis and spermiogenesis.

For 22 dpp control germ cells, the most significant C2 gene set relationships (FDR < 0.1) were Iglesias_E2F_targets_UP (NES −2.44), Nagashima_EGF Signaling_UP (NES −2.31), and for C5 gene sets were RNA processing (NES −2.00), RNA splicing (NES −2.00), mRNA processing (NES −1.92), and RNA binding (NES −1.77). At 23 dpp, the top gene sets enriched for control germ cells in the C2 curated set were PID_Integrin1 pathway (NES −2.19), Hernandez_Mitotic arrest_by_Docetaxel_1_UP (NES −2.08) and PID_Integrin3 pathway (NES −2.06). For C5 gene sets, enrichment in 23 dpp control germ cells was seen for Extracellular Matrix (NES −1.95), Regulation of MAP KINASE ACTIVITY (NES −1.94) and Transmembrane Receptor Protein Kinase Activity (NES −1.77). The 22 and 23 dpp control germ cell gene set relationships determined by GSEA, though differing in certain specifics, were both related to cellular signaling.

### Signaling through ERK1/2 is reduced in *Mgat1* cKO germ cells

**Figure 6:**
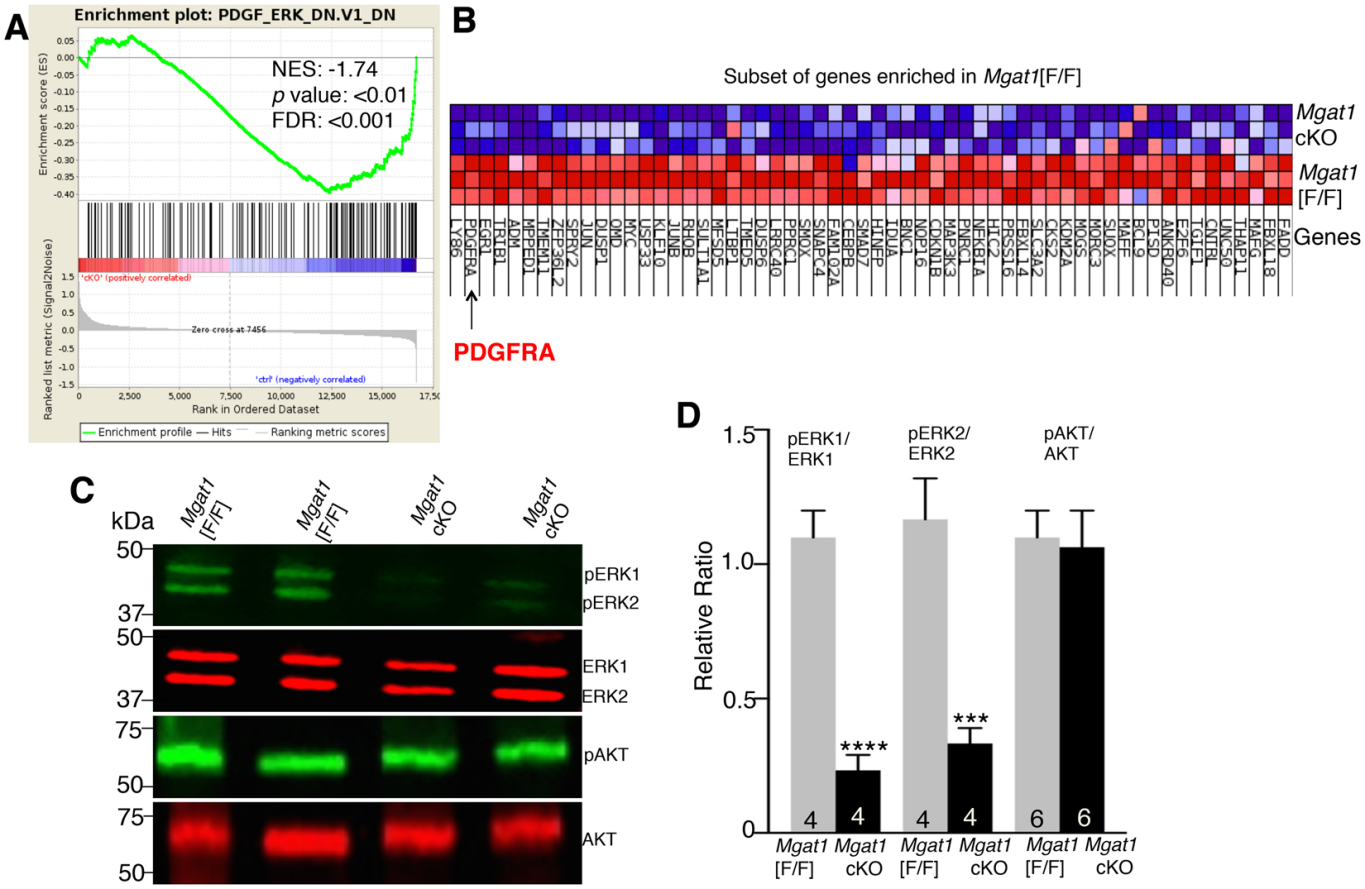
Signaling pathways in *Mgat1* cKO germ cells at 22 dpp. (**A**) GSEA analysis showing enrichment of a PDGF_ERK signature in control germ cells. (**B**) Heat map shows the cluster of DEGs in the PDGF_ERK signaling pathway positively-enriched in control versus *Mgat1* cKO germ cells. Arrow identifies PDGFRA as down-regulated in *Mgat1* cKO germ cells. **(C)** Western blot analysis of phosphorylated and unphosphorylated ERK1, ERK2 and AKT in germ cells of *Mgat1* cKO compared to control. The gels from which these data were obtained are shown in Supplementary Fig. S7. (**D**) Histogram of 2–3 independent experiments analyzed in 4–6 gels is shown (mean ± SEM; ***p < 0.005, ****p < 0.001).

GSEA analysis showed that one of the most significant oncogenic signatures (C6) is PDGF ERK (FDR < 0.001), which was enriched in control versus *Mgat1* cKO germ cells at 22 dpp (Fig. 6A). The heat map shows core enrichment genes that affect the pathway that were up-regulated in control germ cells, including PDGFRA, which regulates ERK1/2 signaling (Fig. 6B). Microarray data and qRT-PCR identified PDGFRA as well as EGFR as down-regulated genes in *Mgat1* cKO germ cells at 22 and 23 dpp (Fig. 2D). Consistent with this, western blot analysis showed that at 22 dpp, pERK1/ERK1 and pERK2/ERK2 levels were markedly reduced in *Mgat1* cKO germ cells compared to controls. However, pAKT/AKT levels were not significantly altered by the loss of *Mgat1* (Fig. 6C,D; Supplemental Fig. S7).

### Basigin signaling is reduced in *Mgat1-*null Lec1 CHO cells

Basigin is a germ cell glycoprotein with complex N-glycans, and the efficient deletion of *Mgat1* in spermatogonia generates basigin with oligomannosyl N-glycans^3^ (Fig. 2A). Deletion of basigin causes the complete loss of *Griffonia simplicifolia* II (GSA) lectin binding to germ cells^10^, suggesting that basigin is a major carrier of germ cell complex N-glycans. Thus, the phenotype of *Mgat1* cKO males may arise, in part, from functional defects in signaling by basigin carrying oligomannosyl, rather than complex, N-glycans. To investigate this hypothesis, we examined ERK signaling induced by mouse basigin in Chinese hamster ovary (CHO) cells that express complex N-glycans, compared to Lec1 *Mgat1*-null CHO mutant cells. The Lec1 CHO mutant^5^ is a cell-based model for *Mgat1* cKO germ cells that lacks MGAT1 activity and expresses only oligomannosyl N-glycans on glycoproteins^6^. We also investigated the expression of connexin 43 (Cx43), a component of the blood-testis-barrier (BTB). The BTB is altered in basigin-null testes^10^.

**Figure 7:**
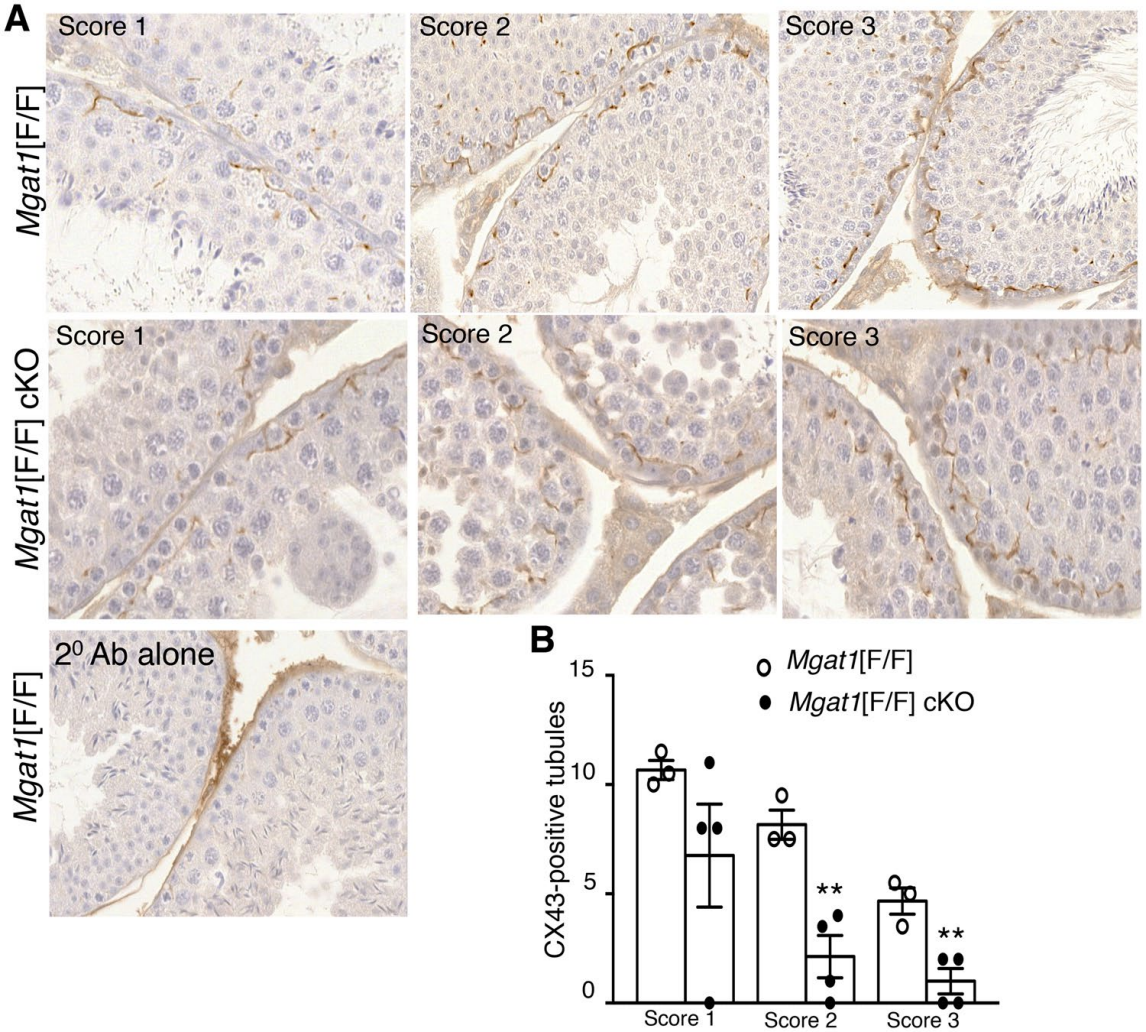
CX43 in 28 dpp control and *Mgat1* cKO testes. (**A**) IHC of CX43 expressed in 28 dpp control and *Mgat1* cKO testis sections were scored blindly as score 1, score 2, or score 3 to reflect poor, medium or high staining, respectively. (**B**) Scores from 90 CX43-positive tubules were counted per genotype. Mean ± SEM, **p < 0.01.

Cx43 immunoreactivity was observed along the basal compartment of seminiferous tubules between spermatogonia and spermatocytes of 28 dpp control and *Mgat1* cKO testis sections, in a pattern typical of BTB proteins (Fig. 7). Immunoreactivity was scored blindly as high (score 3), medium (score 2), or low (score 1). There was a significant reduction in score 2 and score 3 staining in *Mgat1* cKO sections, suggesting that the BTB may be compromised in *Mgat1* cKO testis.

**Figure 8:**
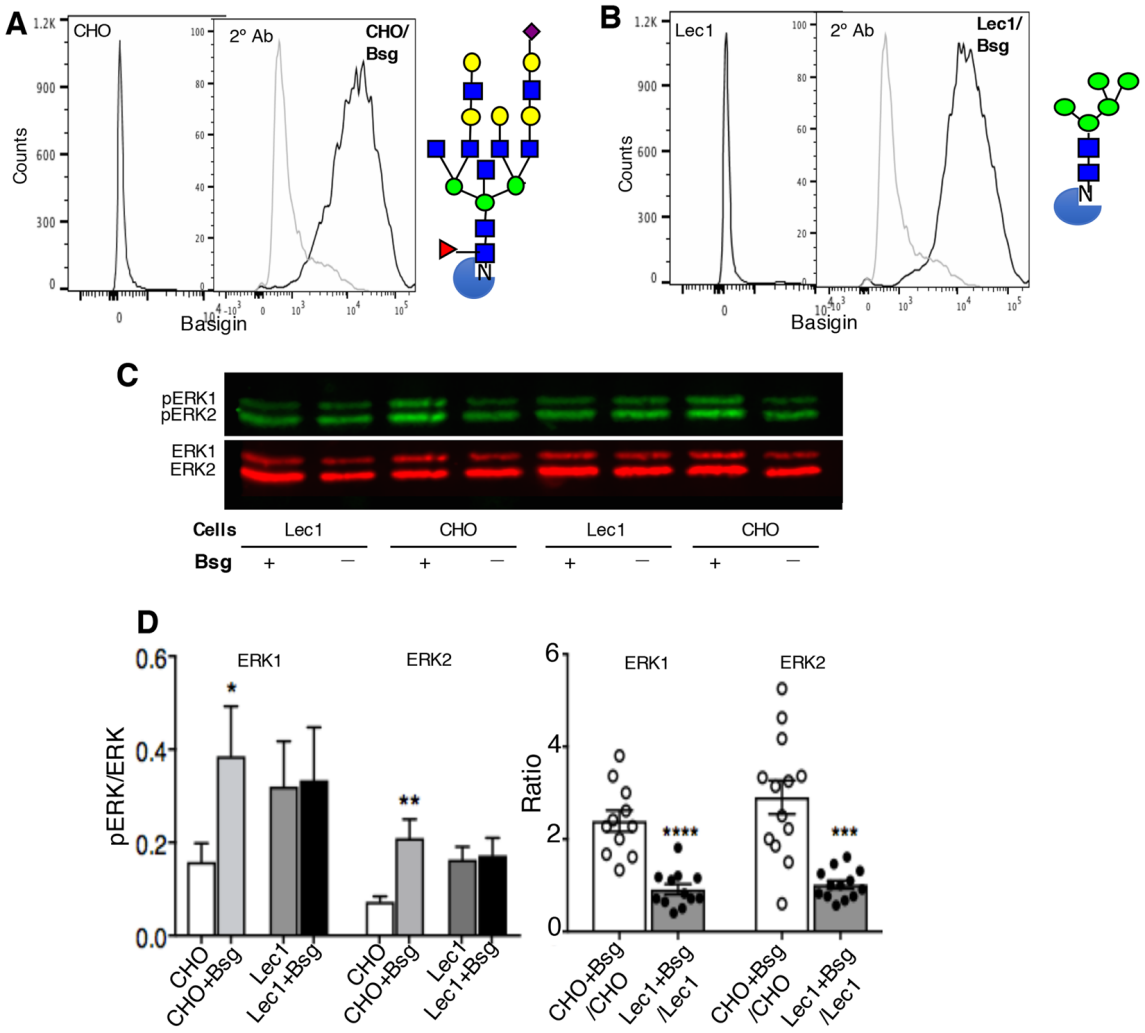
Basigin signaling is reduced in Lec1 CHO cells, a model for *Mgat1* cKO germ cells. Flow cytometry profiles of CHO wild type (**A**) and Lec1 mutant (**B**) CHO cells and basigin transfectants. Also shown is a complex N-glycan typical of CHO cells and the oligomannosyl N-glycan substrate of MGAT1 that accumulates on glycoproteins of Lec1 CHO cells. Blue square, GlcNAc; green circle, Man; yellow circle, Gal; red triangle, fucose; purple diamond, sialic acid. (**C**) CHO wild type, Lec1 and basigin transfectant lysates following 24 hr serum starvation and a 15 min incubation in serum-free medium were analyzed by western blotting using ERK Abs as shown. The gels from which these data were obtained are shown in Supplementary Fig. S9. (**D**) The pERK1/ERK1 and pERK2/ERK2 values for CHO, Lec1 and respective basigin transfectants (left panel), and the ratio of these values in basigin expressing versus non-basigin expressing CHO and Lec1 cells (right panel). Data are mean ± SEM from 9–13 gels of lysates run in 6 independent experiments. *p < 0.05 in (**D**) left panel is based on a one-tailed, unpaired Student’s t test with Welch’s correction; in (**E**, left panel) significance is based on the non-parametric, two-tailed Wilcoxon matched-pairs signed rank test; in (**E**) right panel it is based on the unpaired, two-tailed Student’s t test with Welch’s correction. **p < 0.01, ***p < 0.005 and ****p < 0.001.

There are numerous ligands for basigin (including basigin itself and the soluble extracellular domain of basigin), that induce ERK1/2 phosphorylation^15^. To determine if the nature of the N-glycans on basigin could affect basigin signaling, CHO and Lec1 cells were co-transfected with a mouse basigin cDNA, and a plasmid encoding neomycin resistance. G418-resistant transfectants were sorted for high expression of basigin on the cell surface (Fig. 8A,B). Sorted populations of CHO and Lec1 cells expressing equivalent amounts of cell surface basigin, were serum-starved for 24 hr, and medium was replaced with serum-free medium with and without potential ligands, including cyclophilin A (CypA 100, 250 or 500 ng/ml), or 10% fetal calf serum, or 10 μg/ml anti-basigin Ab. After incubation for 15 min at 37 °C, cells were washed and lysates prepared for western blot analysis. Surprisingly, the inclusion of CypA, FCS or anti-basigin Ab in serum-free medium did not consistently stimulate signaling in CHO or Lec1 basigin transfectants (Supplementary Fig. S8 and not shown). By contrast, activation of ERK1/2 was observed in CHO cells expressing basigin (CHO + Bsg) compared to control CHO cells (CHO) after incubation in serum-free medium alone (Fig. 8C,D; supplemental Fig. S9). The “ligand” in this case may be homomeric interactions between basigin on adjacent cells^15^. Lec1 cells exhibited somewhat higher pERK1/2 levels compared to the CHO cells from which they were derived^5^. However, in contrast to CHO + Bsg cells, signaling was not further stimulated by the presence of basigin in Lec1 + Bsg cells (Fig. 8E,D). A significant difference between CHO and Lec1 cells expressing basigin was apparent when the ratios of CHO + Bsg/CHO and Lec1 + Bsg/Lec1 were compared for ERK1/2 (Fig. 8D). Thus, the combined data are consistent with the hypothesis that basigin carrying oligomannosyl N-glycans signals less well than basigin carrying complex N-glycans.

## Discussion

In this paper we investigated mechanisms that lead to defective spermatogenesis in *Mgat1* cKO males following *Mgat1* deletion in spermatogonia at 3 dpp. Morphological changes began to appear at 24–25 dpp in a small proportion of mutant tubules. About 14% of tubules showed spermatid MNC by 28 dpp. Sertoli cell, spermatogonia and spermatocyte numbers per round tubule were unaffected in *Mgat1* cKO testes at the same stage. To obtain insights into early events that might be the basis of defective spermatogenesis in *Mgat1* cKO males, we interrogated gene expression changes in morphologically normal *Mgat1* cKO germ cells from 22 and 23 dpp males. Surprisingly, we discovered that many genes involved in later stages of spermatogenesis and spermiogenesis were up-regulated in 22 and 23 dpp *Mgat1* cKO germ cells. The majority of up-regulated genes (>85%) encode transcripts that promote spermatogenesis. Therefore, the loss of *Mgat1* in spermatogonia at 3 dpp leads to mutant germ cells at 22 and 23 dpp in which genes normally turned on much later in spermatogenesis, are prematurely up-regulated.

To gain insights into the repertoire of genes connected with functions of MGAT1 in germ cells, we applied molecular network analyses using IPA, GO and GSEA. IPA identified sperm motility as the top canonical pathway with a positive activation Z score, and cAMP signaling as the top canonical pathway with a negative activation Z score. Top biological functions in both 22 and 23 dpp *Mgat1* cKO germ cells were cellular function and maintenance, and reproductive system. Top upstream regulators were TAF7L and FIGLA. *Taf7l null* males have a post-meiotic block in spermiogenesis and are sterile^16^. This block occurs beyond the stage at which spermatogenesis is disrupted in *Mgat1* cKO males. FIGLA functions in oocytes to suppress male germ cell genes involved in spermatogenesis and spermiogenesis^17^. YBX2/MSY2 is a germ cell specific RNA and DNA binding protein of the cytoplasm, most highly expressed in round spermatids during spermatogenesis. Deletion of *Ybx2/Msy2* leads to incomplete nuclear condensation in spermatids, and a block in spermatogenesis^18,19^. The poly(A) polymerase PAPOLB/TRAP polyadenylates a subset of transcription factors and other mRNAs in the cytoplasm. It is expressed in round spermatids and is required for spermatogenesis^20^. Top disease processes Teratozoospermia and Asthenozoospermia were identified as being inhibited. This suggests that the spermatogenesis-specific genes up-regulated in *Mgat1* cKO germ cells normally guard against the development of both these fertility diseases in men. Consistent with this interpretation, GSEA analysis revealed gene enrichment in *Mgat1* cKO germ cells overlapping most significantly with gamete generation, Matzuk spermatid differentiation and Matzuk spermatozoa formation. In summary, the complement of genes up-regulated in 22 and 23 dpp *Mgat1* cKO germ cells suggest that germ cells that lack *Mgat1* are attempting to differentiate prematurely by up-regulating genes involved in spermatogenesis and spermiogenesis. This means that MGAT1 and complex N-glycans on germ cell glycoproteins function during normal spermatogenesis to control differentiation by retarding the expression of genes early in spermatogenesis that are required for later stages of spermatogenesis and spermiogenesis.

The mechanism(s) by which MGAT1 and complex N-glycans on glycoproteins regulate gene expression must necessarily be indirect. It is well established in cell-based experiments that loss or reduced branching of complex N-glycans leads to reduced cell surface residence time of glycoprotein receptors due to the weakening of their interactions with a galectin lattice^21–23^. On this basis it would be predicted that growth factor signaling should be reduced in *Mgat1* cKO germ cells. In fact, our microarray and qRT-PCR data showed that transcripts of both the EGF and PDGF receptors (*Egfr* and *Pdgfra*) were markedly reduced in *Mgat1* cKO germ cells, and the top IPA network identified ERK1/2 as regulating numerous genes in *Mgat1* cKO germ cells. Thus, on several counts, we predicted that growth factor signaling should be reduced in *Mgat1* cKO germ cells. Western blot analysis showed that pERK1/2 levels were indeed markedly reduced in *Mgat1* cKO germ cells. The MAP kinase pathway that leads to ERK1/2 activation is active in spermatogonia and primary spermatocytes and diminished in pachytene spermatocytes^24–26^. Interestingly, pAKT levels were not reduced, consistent with the fact that the block in spermatogenesis in *Mgat1* cKO males is distinct from that in males lacking AKT which is essential for survival and proliferation of pre- and post-meiotic cells^27^. When signaling via basigin was investigated in CHO versus Lec1 cells that lack *Mgat1*, pERK1/2 levels were enhanced by the presence of basigin in CHO but not Lec1 cells. Basigin is a substrate of MGAT1^3^, and is a major carrier of complex N-glycans of germ cell glycoproteins^10^. Basigin has potential ligands in testis that might lead to activation of ERK1/2 in germ cells^15^ including basigin itself and soluble basigin extracellular domain^28^. Importantly, the spermatogenesis defect in basigin-null males is similar to that in *Mgat1* cKO males^10^. The combined data suggest that defective signaling via ERK1/2 due to the loss of complex N-glycans on basigin leads, along with other factors that reduce ERK1/2 signaling in *Mgat1* cKO germ cells, to the block in spermatogenesis in germ cells lacking *Mgat1*. Testing this hypothesis forms the basis of future experiments in males conditionally lacking basigin in germ cells, and related mouse models.

## Methods

### Animals

*Mgat1*[F/F]:Stra8-iCre (mutant) and *Mgat1*[F/F] (control) mice were generated as described^3^. Mice were genotyped by PCR of tail genomic DNA or germ cell DNA as described^3,11^. All mice were bred within the Institute for Animal Studies. The Albert Einstein Animal Institute Committee approved experimental protocols. All methods were performed in accordance with the relevant guidelines and regulations. Mice were sacrificed by carbon dioxide asphyxiation and cervical dislocation. Testes were dissected free of surrounding tissue and weighed.

### Antibodies

Antibodies were rabbit SOX9 pAb (#sc20095, Santa Cruz Biotechnology, Inc., Dallas, TX, USA); rabbit SYCP3 pAb (#NB300–230, Novus Biologicals, Littleton, CO, USA); mouse PCNA mAb PC10 (#P8825, Sigma Aldrich, St. Louis, Mo, USA); rat anti-mouse basigin mAb OX114 (#B3663, LSBio, Inc., Seattle, WA, USA,); mouse connexin 43 mAb CX-1B1 (#13–8300, Invitrogen Corp, Camarillo, CA, USA); rabbit ERK1/2 mAb C33E10, (#3192), mouse pERK1/2 mAb L34F12 (#4696), mouse AKT mAb 40D4 (#2920) and rabbit pAKT mAb D9E (#4060) were from Cell Signaling Technology, Danvers, MA, USA; horse radish peroxidase(HRP)-conjugated goat anti-rabbit IgG (#65–6120, Invitrogen Corp) and goat anti-rat IgG (#112–005–003, Jackson Immunoresearch Laboratories, Inc., West Grove, PA, USA); LI-COR fluorescent secondary Abs (#P/N 925–32211, goat anti-rabbit IgG IRDye 800CW; #P/N 925–6802, goat anti-mouse IgG IRDye 680LT; LI-COR Biotechnology, Lincoln, NE, USA); and biotinylated goat anti-mouse IgG (#BA-9200).

### Histology and Immunohistochemistry

Testes from 15–28 dpp males were fixed in Bouin’s fixative (#100503–962, Electron Microscopic Sciences, Radnor, PA, USA) for 48 hr at room temperature (RT) and processed by the Einstein Histotechnology and Comparative Pathology Facility. Testes were paraffin-embedded. Serial sections (5 μm) were collected on positively-charged slides and stained with H&E or the PAS reagent. Unstained sections were used for IHC. Slides were microwaved in 10 mM citrate buffer (pH 6.0), incubated in 3% hydrogen peroxide in methanol (15 min, RT) and blocked in 5% goat serum (#G9023, Sigma-Aldrich) in phosphate-buffered saline (pH 7.2) containing 1 mM CaCl_2_, 1 mM MgCl_2_ and 0.05% Tween 20 (PBST) for 60 min at RT. Sections were incubated overnight at 4 °C in blocking buffer containing anti-CX43 (1:200) or 1:500 anti-SOX9, anti-SYCP3 or anti-PCNA Abs in Tris-buffered saline, pH 7.2 (TBS). Secondary antibody alone and peptide inhibition were the negative controls. After washing with PBST (no cations), sections were incubated with biotinylated secondary Ab (60 min, RT; 1:500 in PBST (CX43); 1:1000 in TBS (SOX9, SYCP3 and PCNA), detected using ABC Vectastain (#PK-6100, Vector laboratories, Burlingame, CA, USA) and 3,3′diaminobenzidine (#SK-4100, Vector Laboratories) followed by counterstaining with haematoxylin (#MHS-16, Sigma-Aldrich). Slides were observed by light microscopy (Leica Microsystem, Wetzlar, Germany) and scanned using a Perkin Elmer P250 high capacity slide scanner (3D Histech P250 high capacity slide scanner, Perkin Elmer, Waltham, MA, USA).

### Germ cell isolation and extraction

Testes were collected in DMEM/F12 (Gibco Life Technologies Corp., Grand Island, NY, USA) on ice, detunicated, and germ cells were isolated as described^29^. Briefly, testes were minced in 10 ml solution 1 (DMEM/F12 containing 500 μg/ml collagenase 1 A (#CO130–1G) and 200 μg/ml DNase I (#DN25–100MG) for 10 min in a shaking water bath, tubules were allowed to sediment in Hank’s buffered saline (HBSS, #55–022–PB, Corning, Manassas, VA, USA) containing 5% Percoll (#17–0891–01, GE Health Care Bio-Sciences, Uppsala, Sweden) in water for 20 min, followed by incubation in 10 ml solution 2 (DMEM/F12 containing 1.25 mg/ml trypsin (#T4799–5G) and 200 μg/ml DNase I), filtered through 40 micron nylon mesh, and washed in PBS. All enzymes were from Sigma-Aldrich. DNA and protein were extracted from germ cells of one testis and RNA from the other. DNA was isolated by the DNeasy Blood & Tissue Kit (#69504, Qiagen, Germantown, MD, USA), protein by homogenization in 200 μl 1.5% Triton X-100 with protease inhibitor (#05 892 791 001; Thermo Fisher Scientific, Springfield Township, NJ, USA) by pellet pestle (#749540–000, Kontes Glass Co, Vineland, NJ, USA) and RNA by the All-Prep RNeasy mini kit (#74104) and RNeasy mini-elute cleanup kit (#74204) from Qiagen. After centrifugation (3000 rpm, 10 min, 4 °C), protein was determined by Bradford assay (Bio-Rad, Hercules, CA, USA). RIN was assessed using an Agilent 2100 Bioanalyzer (Agilent Technologies, Santa Clara, CA, USA) and measured using a Nanodrop ND1000 Spectrophotometer. RNA and protein were extracted from 23 dpp germ cells using Trizol Reagent (Sigma Aldrich).

### Endoglycosidase H digestion

Lysate containing 20–60 μg testis protein was treated with 5 mU Endo H from *S*. *plicatus* (#11088726001, Roche Diagnostics, Manheim, Germany) or water in 20 μl manufacturer’s buffer at 37 °C for 2 hr. Reactions were stopped by adding SDS gel loading buffer and heating at 95 °C, 10 min.

### Microarray analysis

Testis RNA (150 ng, RIN ≥ 9) was provided to the Genomics Core Facility of the Albert Einstein College of Medicine for conversion to cDNA, labeling and hybridization to Affymetrix GeneChip™ Mouse Gene 2.0 ST Array (Affymetrix, Santa Clara, CA, USA). After gene level normalization, signal summarization and background subtraction, raw intensity data (.CEL files) were transformed to .CHP files using Affymetrix Expression Console software. Genes up- or down-regulated with a fold-change >2.0 or <−2.0 were determined using the Affymetrix Transcriptome Analysis Console. Differences between medians (log_2_) were determined and transformed to linear fold-change. Statistical significance was assessed using ANOVA and FDR *p* values.

### Quantitative PCR

RNA (500 ng) was reverse-transcribed into cDNA using the verso cDNA synthesis kit (#AB1453/B, Thermo Fisher) with an oligo-dT primer according to the manufacturer’s protocol. Real time PCR was performed using Absolute Blue QPCR mix (#AB4162, Thermo Fisher) on a master cycler (ViiA 7, Thermo Fisher). PCR conditions were 95 °C 30 sec, followed by 40 cycles at 95 °C 15 sec, 60 °C 15 sec and 72 °C 20 sec. Gene expression relative to actin (*Actb*) was calculated as log_2_^dCT^, *Mgat1* cKO values were subtracted from control (log_2_^ddCT^) and converted to fold-change.

### Gene Ontology

Gene ontology (PANTHER version 11.1)^30,31^ analysis was used to investigate functions for genes with fold-change ±1.5 and *p* < 0.05. The analysis classifies functions according to biological process, cellular component and molecular function.

### Ingenuity Pathway Analysis

IPA of DEGs was performed using QIAGEN’s Ingenuity Pathway Analysis algorithm (www.qiagen.com/ingenuity, QIAGEN, Redwood City, CA, USA) for genes with fold-change ±1.5 and overlap *p* < 0.05.

### Gene set enrichment analysis

GSEA was performed to determine DEGs that were enriched in gene lists extracted from MSigDB^32,33^ to determine enrichment in gene sets from the curated (C2), GO (C5), and oncogenic signatures (C6) collections.

### Western blot analysis

Protein was extracted from germ cells in 200 μl homogenization buffer (1.5% Triton X-100, protease Inhibitor and PhosSTOP (#04906845001, Roche Diagnostics), centrifuged (3000 rpm, 10 min, 4 °C), and supernatant protein determined. For detection of ERK and AKT, protein (60 μg) was separated on 10% SDS polyacrylamide, transferred to nitrocellulose membrane (#162–0095, Bio-Rad) in 10% methanol, blocked in LI-COR Odyssey blocking buffer (#P/N 927–40100; OBB) 1 hr at RT, and incubated overnight at 4 °C with antibodies in OBB. Membranes washed 3× in PBST and then PBS (both no cations) were probed with LI-COR fluorescent Abs in OBB for 1  hr at RT in the dark. After washing, imaging was performed (LI-COR Odyssey scanner) and band intensity was calculated (LI-COR Odyssey software). For detecting basigin, 40–60 μg protein was separated by SDS-PAGE (10%). After transfer to PVDF membrane (10% methanol) and blocking in 3% fish gelatin (#G7041, Sigma-Aldrich) in TBST, membranes were incubated in anti-basigin mAb (1:1000 in 3% fish gelatin) overnight at 4 °C. The membrane was probed with secondary Ab 1 hr at RT, treated with Super Signal West Pico chemiluminiscent substrate (#34080, Thermo Fisher) and exposed to film.

### ERK activation by basigin in CHO and Lec1 Cells

CHO wild type (Pro-5) and *Mgat1* null Lec1 CHO cells (Pro-5Lec1.3 C)^5^ were co-transfected with 3 μg mouse basigin plasmid cDNA (MC200670, Origene, Rockville, MD, USA) and 0.3 μg pSV2NEO in 6 well plates with Xtreme Gene HP DNA transfection reagent (#06 366 244 001, Sigma-Aldrich) at 1:3, DNA:reagent. Next day the cells were trypsinized and re-plated in a 10 cm plate in Gibco Alpha + MEM medium (#11900–073, Thermo Fisher) containing 10% fetal calf serum (FCS), penicillin, streptomycin and 1.5 mg/ml active G418 (#400–111 P, Gemini, Sunnyvale, CA, USA). Transfectant populations were analyzed by flow cytometry using anti-basigin mAb and anti-rat IgG conjugated to Alexa-Fluor-488. The top 3% of binders were sorted and used in experiments.

To assay ERK activation, cells at ~80% confluency were serum-starved for 24 hr in alpha + MEM medium. To stimulate signaling, medium was replaced with alpha + MEM, or alpha + MEM containing CypA (#3589-CA, R&D Systems), or 10% FCS or 10 μg/ml basigin mAb, for 15 min at 37 °C. After washing, cells were lysed in 100 μl RIPA (#20–188, EMD Millipore, Billerica, MA, USA), 0.1% SDS, protease inhibitor and PhosSTOP (Sigma Aldrich). After centrifugation (3,000 rpm, 5 min, 4 °C), 40 μg protein was electrophoresed on 12% SDS-PAGE gels. Proteins were transferred to nitrocellulose using the Pierce G2 blotter, washed in distilled water, and dried at 4 °C. Membranes were blocked with OBB in TBS (1 hr, RT), incubated in primary mAbs in OBB 0.05% Tween at 4 °C overnight followed, after washing, by LI-COR secondary antibodies for 1 hr at RT in the dark. Blots were washed 4× with TBST, twice with TBS, dried and analyzed as described above.

### Statistical Analysis

Unless otherwise noted, results are presented as mean ± SEM for independent measurements or individual mice. A one-way ANOVA or unpaired, two-tailed Student’s t test with Welch’s correction was used to determine *p* values in Graph Pad Prism 7.0a (Graph Pad Software Inc., La Jolla, CA, USA), unless otherwise noted. Limited amounts of RNA from 22 and 23 dpp germ cells precluded testing sufficient replicates to establish a Gaussian distribution for qRT-PCR data. However, a Gaussian distribution was established by the D’Agostino-Pearson test in Prism for most data sets in Fig. 7D,E. For other samples a Gaussian distribution was assumed and parametric tests of significance were performed, unless otherwise noted.

## Electronic supplementary material

Supplementary Information
