# Author Correction: MGAT1 and Complex N-Glycans Regulate ERK Signaling During Spermatogenesis

**DOI:** 10.1038/s41598-023-30418-0

**Published:** 2023-02-28

**Authors:** Barnali Biswas, Frank Batista, Subha Sundaram, Pamela Stanley

**Affiliations:** 1 Department of Cell Biology, Albert Einstein College of Medicine, New York, NY 10461 USA; 2 Present Address: Biochemistry and Molecular Biology Department, University of Debrecen, Debrecen, Hungary

Correction to: *Scientific Reports* 10.1038/s41598-018-20465-3, published online 31 January 2018

This Article contains errors.

The image in Figure 6D shows an incorrect number of independent germ cell samples analyzed in western blots for ERK and pERK. In the revised Figure 6D, the results from the gel shown in Figure 6C, and 3 additional western blots of germ cell lysates from Control (*Mgat1*[F/F], Mgat1[F/+]:*Stra8*-iCre or *Mgat1*[+/+]) and *Mgat1* cKO males are included.

As a result, the Figure legend for Figure 6D should read:

“(**D**) Histogram of the relative ratio of ERK and AKT signaling determined from western blot analyses of germ cell lysates prepared from the number of mice noted in each bar (mean ± SEM; **p < 0.01). Control mice included *Mgat1*[F/F], *Mgat1*[F/+]:*Stra8-*iCre and *Mgat1*[+/+] 22-day males.”

The correct Figure 6 and its corrected accompanying legend appear below.

**Figure 6:**
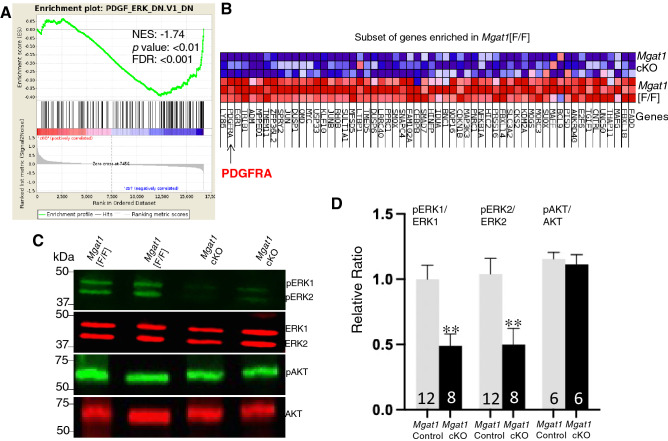
Signaling pathways in *Mgat1* cKO germ cells at 22 dpp. (**A**) GSEA analysis showing enrichment of a PDGF_ERK signature in control germ cells. (**B**) Heat map shows the cluster of DEGs in the PDGF_ERK signaling pathway positively-enriched in control versus *Mgat1* cKO germ cells. Arrow identifies PDGFRA as down-regulated in *Mgat1* cKO germ cells. (**C**) Western blot analysis of phosphorylated and unphosphorylated ERK1, ERK2 and AKT in germ cells of *Mgat1* cKO compared to control. The gels from which these data were obtained are shown in Supplementary Fig. S7. (**D**) Histogram of the relative ratio of ERK and AKT signaling determined from western blot analyses of germ cell lysates prepared from the number of mice noted in each bar (mean ± SEM; **p < 0.01). Control mice included *Mgat1*[F/F], *Mgat1*[F/+]:*Stra8-*iCre and *Mgat1*[+ /+] 22-day males.

Furthermore, some primer sequences are not included in the Methods section, under the subheading ‘Quantitative PCR’. Primer sequences are given in Supplementary Table S4 and as follows:

*Acrv*, (For) TGAAGTTTCGGGTGACGAAGCAGGT: (Rev) GCTGGGAGTTTTGAGTGGTGCATAC;

*Dbil5*, (For) GTACAGCTTTTACAAACAGGCCACCC: (Rev) CCACTTTAGCAATGTAGATCCTCATGGC;

*Cyp11a1*, (For) GTGGACCCCAAGGATGCGTCGATACTCTTC: (Rev) ACCTCTTGGTTTAGGACGATTCGGTC;

*Rhox5,* (For) TCAAGGAAGACTCGGAAGAACAGCAT: (Rev) CACTATCCTTGTCCCCATCACCCATA;

*Actb*, (For) CGGTTCCGATGCCCTGAGGCTC: (Rev) TGTCAGCAATGCCTGGGTACATGGTGGT.”

