# Supplementary material for: MGAT1 and Complex N-Glycans Regulate ERK Signaling During Spermatogenesis
Source: Sci Rep. 2018 Jan 31;8:2022. doi: 10.1038/s41598-018-20465-3 (PMC5792458; doi:10.1038/s41598-018-20465-3)
Supplement: Supplementary file 1 — Supplementary Information [file 41598_2018_20465_MOESM1_ESM.pdf]

Supplementary Information

MGAT1 and Complex N-Glycans Regulate ERK Signaling During Spermatogenesis

Barnali Biswas[1], Frank Batista[1,2], Subha Sundaram[1] and Pamela Stanley[1*]

[1] Department of Cell Biology, Albert Einstein College of Medicine, New York, NY, 10461.

[2] Present address: Biochemistry and Molecular Biology Department, University of Debrecen, Hungary,

Corresponding author: pamela.stanley@einstein.yu.edu

Supplementary Methods

Antibodies. Mouse sp56 mAb 7C5 (#55101, QED Biosciences Inc., San Diego, CA, USA); rabbit TAF7L pAb (#ARP37923_T100, Aviva Systems Biology Corp, San Diego, CA, USA); mouse beta tubulin mAb (#E7, DSHB, Iowa, USA).

Antibody specificity controls. Antibody specificities were investigated using 2º Ab alone and antigen blocking peptides. Sox9 Ab (#sc-166505, Santa Cruz Biotechnology, Inc., Dallas, TX, USA) with and without Sox9 blocking peptide (#sc-166505 P, Santa Cruz Biotechnology) in a ratio of 1:10 (0.2:2 µg) in 2 ml blocking buffer was placed in a shaker at 37ºC for 2 hr at RT, added to testis sections after the blocking step, and incubated overnight at 4ºC. After washing with PBST (no cations), sections were incubated with biotinylated goat anti-mouse IgG (#BA-9200, Vector Laboratories) detected using Vectastain ABC-HRP kit (#PK-4000, Vector Laboratories), 3,3'diaminobenzidine (#SK-4100, Vector Laboratories), and counterstained with haematoxylin (#MHS-16, Sigma-Aldrich). Sycp3 antibody (#NB300-230, Novus Biologicals, Littleton, CO, USA) was used at 1 µg in 200 µl 5% goat serum (#G9023, Sigma-Aldrich, St. Louis, Mo, USA) in phosphate-buffered saline (pH 7.2) containing 1 mM $CaCl_2$, 1 mM $MgCl_2$ and 0.05% Tween 20 (PBST) for 60 min at room temperature. Sycp3 antibody with and without Sycp3 blocking peptide ((#NB300-230PEP, Novus Biologicals) mixed in a ratio of 1:10 µg in 2 ml blocking buffer were shaken at 37ºC for 2 hr at RT, added to testis sections after the blocking step and incubated overnight at 4ºC. After washing with PBST (no cations), sections were incubated with biotinylated goat anti-rabbit IgG (#BA-1000, Vector Laboratories) detected using Vectastain ABC-HRP kit (#PK-4000, Vector Laboratories), 3,3'diaminobenzidine (#SK-4100, Vector Laboratories), and counterstained with haematoxylin (#MHS-16, Sigma-Aldrich).

Immunofluorescence. Five micron sections from 28 dpp males were fixed in Bouin's solution, paraffin embedded and sectioned. The slides were deparaffinised in histo-clear solution (#HS-200, National diagnostics, USA) and rehydrated in a graded series of ethanol. Antigen retrieval was performed in 0.01M citrate for 10 min in a microwave. Slides were rinsed in PBS (pH 7.2, no cations) for 5 min, blocked in 5% BSA (#700-100P, Gemini Bioproducts, CA, USA) in PBS for 2 hr at RT and incubated with mouse anti-sp56 (clone 7C5) mAb. Sections were washed 3 times with PBS (no cations), and incubated in goat anti-mouse IgG secondary antibody conjugated to Alexa fluor 488 (Invitrogen) for 2 hr at RT. After washing 3 times with PBS, sections were mounted with Fluoroshield mounting medium containing 4,6-Diamidino-2-phenylindole (DAPI, #ab104139, Abcam, Cambridge, MA, USA). Sections incubated with secondary antibody alone served as negative control. Images were obtained by fluorescence microscopy (Leica Microsystems, Wetzlar, Germany).

Table S1. Elongated spermatids and MNC in 24 – 28 dpp testis. Elongated spermatids and multinuclear cells (MNC) were identified in testis sections stained by H&E. Values are mean ± SEM from 50 (24 dpp) or 100 (25 and 28 dpp) round tubules counted in 1 testis section from 3-4 males, as indicated. Significance was determined by Student's t test with Welch's correction.

**Intratubular Cells** (No. Mice)	***Mgat1*[F/F]** (n=4)	***Mgat1*[F/F]: Stra8-iCre** (n=4)	***Mgat1*[F/F]** (n=3)	***Mgat1*[F/F]: Stra8-iCre** (n=4)	***Mgat1*[F/F]** (n=3)	***Mgat1*[F/F]: Stra8-iCre** (n=3)
Days Post Partum	24	24	25	25	28	28
Elongated Spermatids	0	0	29 ± 3	10 ± 4 (*p*<0.01)	53 ± 2	6 ± 1 (*p*<0.0001)
MNC	0	2 ± 1 (*p*<0.01)	0	5 ± 1 (*p*<0.01)	0	14 ± 1 (*p*<0.001)

Table S2. Germ cell preparations from 22 dpp mice used for microarray. RNA was isolated from germ cells of one testis, and protein and DNA were extracted from the other, as described in ***Methods.***

Mouse	Body Weight (g)	Left Testis Weight (mg)	Right Testis Weight (mg)	Germ Cells per Testis (X 10^4)	Protein per Testis (µg)	RNA per Testis (µg)	RNA Integrity Number (RIN)
***Mgat1*[F/F]**							
SCM1001	13.0	27.3	30.1	62	240	4.0	9.4
SCM1002	13.9	39.0	28.8	85	180	5.2	9.2
SCM1003	13.5	33.9	33.6	64	160	4.0	9.3
***Mgat1*[F/F]: Stra8-iCre**							
SCM981	12.1	29.2	34.0	116	120	4.5	9.1
SCM982	13.6	30.1	32.3	75	180	3.0	9.2
SCM983	12.2	29.4	32.7	92	132	4.2	9.4

Table S3. DEGs in *Mgat1* cKO versus control germ cells

Probe Name	Gene Symbol	Fold-Change 22 dpp	Fold-Change 23 dpp
17328107	*Tnp2*	23.09	9.92
17328113	*Prm2*	16.06	5.44
17224191	*Tnp1*	15.23	6.64
17413507	*Ccin*	13.75	6.68
17477288	*Klk1b7-ps*	13.10	28.23
17321199	*H1fnt*	12.82	9.32
17407386	*Lelp1*	11.07	8.54
17410617	*Dapp1*	10.73	0.88
17215016	*Spata3*	10.06	5.47
17426944	*Fam154a*	9.69	11.11
17319484	*Dnajb7*	9.17	5.83
17336824	*Hspa1l*	8.38	8.60
17396192	*Cypt12*	8.36	4.27
17494303	*Ubqlnl*	8.35	2.75
17515517	*Cypt4*	8.33	2.20
17546412	*Rbm31y*	8.19	3.96
17466216	*Prss37*	8.18	4.07
17346744	*Txndc2*	8.14	10.46
17312350	*Spatc1*	7.67	2.65
17487361	*Apoc2*	0.29	0.95
17293858	*Zfp640*	0.09	0.83
17230045	*Ifi204*	0.11	0.69
17350982	*Cd74*	0.23	0.77
17225169	*Snora75*	0.25	0.81
17278253	Serpina3a	0.28	1.21
17539536	*Figf*	0.29	0.79
17372912	*Olfr1188*	0.15	1.00

Fold-change is transformed $\log_2$ *Mgat1* cKO compared to control (p <0.05)

Table S4. Primers used in quantitative real time PCR.

Gene	Forward 5'- 3'	Reverse 5'- 3'
Akap1	GCTCCGGAGACAAAGCTATGA	ATTCAGTGGATAGCTGTTGCGG
Akap3	GACACCGTTCAGAACAAGCAACTG	CTGGATGAAGCAGTGGGAAATGTC
Akap4	AGAGGCGTGTGCAAGGTAGAT	CCAGGTCAGAAGGCGAGTTAAATCT
Gfra2	CTGCCAGATGACCTCAGTGATAG	GCTCACGACAAATATCAGCCCAG
Figf	AGCGAACATGGACCAGTGAAGGA	AGCTTCTAGTTTGGAGGAGTTGCTG
Camk4	AGCTGGTCACAGGAGGAGAA	TCTTTATGCAACTCCAGCCCC
Hsfy2	GGATGAGGACGGGACTTACA	GCTTCTCTCCCCGACTTTC
Spert	ACAACACAGGAGGACTCGAAGACACT	AGGATGGCAAAGTAGGCCCTAACCTAC
H1fnt	CACAGGAGTTTGTCAGTGCCAAAGAGCA	TACGGCCTACAAAGGAAGACACCAGTCCTA
Tbc1d21	CGTGGTTCTGCCTTTGCTTC	TGGCCTACAGCATGCTACAG
Fam1541	TGCTACCGGAAACCATCTGCACCAAT	CGATTTTAACTACCTCCCTACCTGTCC
Sycp3	CATTCTGGGAAATCTGGGAAGCCACCT	CCAGCATATTCTGTACTTCACCTCCAAC
Iqcf3	GTTCGTTAAACCTAAACCTAAAGTCCA	CAACCAGAGTTCCACATTGAAATCCT
Hsfy2	GGATGAGGACGGGACTTACA	GCTTCTCTCCCCGACTTTC
Tnp2	TCTCGACACTCACCTGCAAGA	CTTGTATCTTCGCCCTGAGCTA
Spem1	CTGCTCTTCGCCCTGAGCTA	AAGATGGACTTTGGGTCTTATCGTCT
Prm2	CTCGTAAGAGGCTACATAGGATCCAC	TGCCTCCTACATTTCCTGCACCT

Spata3	AAACCTGCCTCATCTCCGTTCCTGGTA	AGTCCCGAGGGTAGGAGGACATCAAA
Ccin	ATTGGGCTTGTCTTCCACACCATG	TACAGGTTCCGGTCACCCTTAGT
Klk1b7	ATGAGGAGTGTGCCAAAACC	CCATGGAGAACACCATCACA
Fam154a	TGCTACCGGAAACCATCTGCACCAAT	CGATTTTAACTACCTCCCTACCTGTCC
H1fnt	CACAGGAGTTTGTCAGTGCCAAAGAGCA	TACGGCCTACAAAGGAAGACACCAGTCCTA
Il1b	GGGCCTCAAAGGAAAGAATCTATACC	CTGATGTACCAGTTGGGGAAC
Olfr1188	CTTGCTACAACTTGCCTGCACTGA	CCTGCAGTGCTATGTGTTCTCAA
Serpina3n	AGCCTGGAGGATGTCCTTTCAA	GATCATTATCAGGAAAGGCCCATTG
Zfp640	GATGCAGTGACTTTTGACGATGTGC	GGTTCTTCAATGTGATGGTCTTCCC
Pdgfra	AGTGTTGGTGCTGTTGGTGATTGTC	TTCCCATCTGGAGTCGTAAGGCAA
Egfr	GGCGTTGGAGGAAAAGAAAG	TTCCCAAGGACCACTTCACA
Rarres	AAGACTCCTCTGCACAGCATCA	TGAGCTGGACCAAGTAATAGTGCA
Tnp1	ATGGCATGAGGAGAGGCAAGAA	CCGCAACAGCTATCCACTGAAT
Taf7l	GGGAAGTCGTTGATGATGATGATGC	GATGTGTTTCGGGAGATGATGGG
Taf7l	ATCATTCAGCCGTCCTTGT	GAAGGTCAAATTGAAGGCCA
Mgat1	CTTCACCCAGTTGGACCTGT	GCCTTGAAGCTGTCTCTGCT

Table S5. Significantly enriched gene sets in *Mgat1* cKO germ cells at 22 dpp by GSEA

**Name**	**No. Genes in Set**	**ES**	**NES**	**Nom *p* value**	**FDR q value**	**Msig DB**
MATZUK__SPERMATID DIFFERENTIATION	34	0.71	2.09	0.0	0.00	C2
MATZUK_SPERMATOZOA	108	0.51	1.85	0.0	0.08	C2
WEBER_METHYLATED_LCP_IN_SPERM_UP	15	0.97	2.42	0.0	0.00	C2
SU_TESTIS	68	0.50	1.67	0.0	0.25	C2
WEBER_METHYLATED_HCP_IN_SPERM_UP	19	0.65	1.69	0.0	0.23	C2
NAGASHIMA_EGF_SIGNALING_UP	56	-0.62	-2.31	0.0	0.0	C2
cAMP_UP.V1_UP	178	-0.38	-1.74	0.0	0.00	C6
mTOR_UP.V1_DN	174	-0.39	-1.79	0.0	0.00	C6
PDGF_ERK_DN.V1_DN	134	-0.39	-1.74	0.0	0.00	C6
E2F1_UP.V1_DN	177	-0.43	-1.92	0.0	0.0	C6
CELL_CYCLE_CHECKPOINT	45	-0.52	-1.86	0.00	0.07	C5
DNA_REPLICATION	95	-0.43	-1.76	0.0	0.11	C5
GAMETE_GENERATION	100	0.56	2.00	0.0	0.00	C5
GENERATION_OF_A_SIGNAL_INVOLVED_IN_CELL_CELL_SIGNALING	28	0.65	1.81	0.00	0.06	C5
AKT_UP.V1_UP	161	0.36	1.36	0.04	0.26	C6

GSEA, Gene Set Enrichment Analysis; ES, enrichment score; p **<**0.05; false discovery rate (FDR), **<**0.25; NES, normalized enrichment score; C2, curated gene sets; C5, gene ontology (GO) gene sets; C6, oncogenic signatures gene sets.

Supplementary figure legends

Figure S1. Acrosomal marker sp56 reveals MNC containing spermatids. Immunofluorescence microscopy for sp56 in a testis section of a 28 dpp *Mgat1* cKO mouse (representative of 40 tubules per 2 sections from 3 control and 3 *Mgat1* cKO males). Arrows indicate MNCs. The negative control was 2º Ab alone. DAPI was used to stain the nucleus.

Figure S2. Negative controls for antibodies used in Fig. 1B. IHC for Sox9 (Sertoli cells) and Sycp3 (spermatocytes) from 25 dpp control males (each representative of 2 entire sections). Negative controls were 2^0 Ab alone and Sox9- or Sycp3-blocking peptide combined with 1º Ab. IHC for PCNA (spermatogonia) at 25 dpp. Negative control was 2^0 Ab alone (representative of 2 testis sections). Images were photographed at 20X.

Figure S3. Properties of germ cells used in microarray analyses. (A) Quantitative RT-PCR (qRT-PCR) of cell-type specific genes was performed in duplicate to test for the presence of Sertoli cells (*Rhox5*), Leydig cells (*Cyp11a1*), round spermatids (*Dbil5*), spermatocytes (*Sycp3*) and late spermatids (*Acrv*) in 22 dpp whole testis (n=2 mice, 4 values) versus germ cells (n=3 mice, 6 values). Relative expression compared to *Actb* was normalized to testis (mean ± SEM based on the Student's t test with Welch's correction; *$p<0.05$). The data show that germ cell preparations contained low levels of non-germ cells. **(B)** *Mgat1* transcripts from 22 or 23 dpp germ cells from control (n=3, 6 values) and *Mgat1* cKO (n=3, 6 values) mice were quantitated by qRT-PCR in two experiments performed in duplicate. Relative expression compared to *Actb* is shown (mean ± SEM based on the Student's t test with Welch's correction; *$p<0.05$, ***$p<0.001$). **(C)** PCR of genomic DNA using two primer sets to detect wild type and floxed alleles or deleted alleles respectively in control and *Mgat1* cKO germ cells at 22 dpp. The lack of PCR products from wild type alleles shows that germ cells contained few non-germ cells in the preparations. **(D)** Western blot analysis for basigin before and after endoglycosidase H treatment of germ cell lysates at 22 and 23 dpp. Full length gels from which the gel portions shown in Fig. 2A were obtained.

Figure S4. Gene expression changes in 22 and 23 dpp control versus *Mgat1* cKO germ cells. Volcano plots of DEGs plotted as fold-change (linear) versus the −10 log10 of the adjusted *p* value (FDR $p<0.05$, ANOVA $p<0.05$) are shown for **(A)** 22 dpp and **(B)** 23 dpp microarray data. Thresholds are shown as dashed lines. Genes up-regulated >2-fold are red and down-regulated <-2-fold are green.

Figure S5. GO analysis of DEGs from 22 dpp microarray data. DEGs in 22 dpp *Mgat1* cKO germ cells determined by PANTHER version 11.1 are shown. The classification of enriched genes in the *Mgat1* cKO dataset based on GO terms **(A)** Biological Process, **(B)** Molecular Function, and **(C)** Cellular Component, are given. The X axis shows the number of genes present in our dataset and the Y axis shows the top processes in each function. The enrichment score for each process is shown beside the bar graph; +/- shows whether the process is over (+) or under (-) enriched.

Figure S6. TAF7L in germ cells of 23 dpp control and *Mgat1* cKO males. **(A)** IHC for TAF7L at 23 dpp (representative of 2 sections from 3 control and 3 *Mgat1* cKO mice). The negative control was 2º Ab alone. **(B)** qRT-PCR analysis of *Taf7L* transcripts from control (n=4) and *Mgat1* cKO (n=5) germ cell preparations at 23 dpp compared to two house-keeping genes *Actb* and *Rps2*. The values from individual mice with each house-keeping gene are mean ± SEM. *$p<0.05$. **(C)** Western blot analysis of TAF7L in germ cell lysates of *Mgat1* cKO (n=3) compared to control (n=3) males at 23 dpp. **(D)** Histogram of two independent experiments analyzed in 4-6 gels is shown (mean ± SEM).

Figure S7. Signaling pathways in control and *Mgat1* cKO germ cells at 22 dpp. Western blots of pERK1/2, ERK1/2, pAKT and AKT. Full length gels from which data in Fig. 6 were obtained.

Figure S8. Effects of CypA on basigin signaling via ERK1/2. CHO and Lec1 cells were serum-

starved for 24 hr and then treated for 15 min in fresh serum-free medium containing 250 ng/ml CypA. ERK1/2 and pERK1/2 were quantitated in western blots and are plotted as mean +/- SEM of the ratio of basigin-expressing cells incubated with and without CypA. There were no significant differences.

Figure S9. Basigin is a functional target of MGAT1. Western blots of pERK1/2 and ERK1/2. Full length gels from which data in Fig. 8 were obtained.

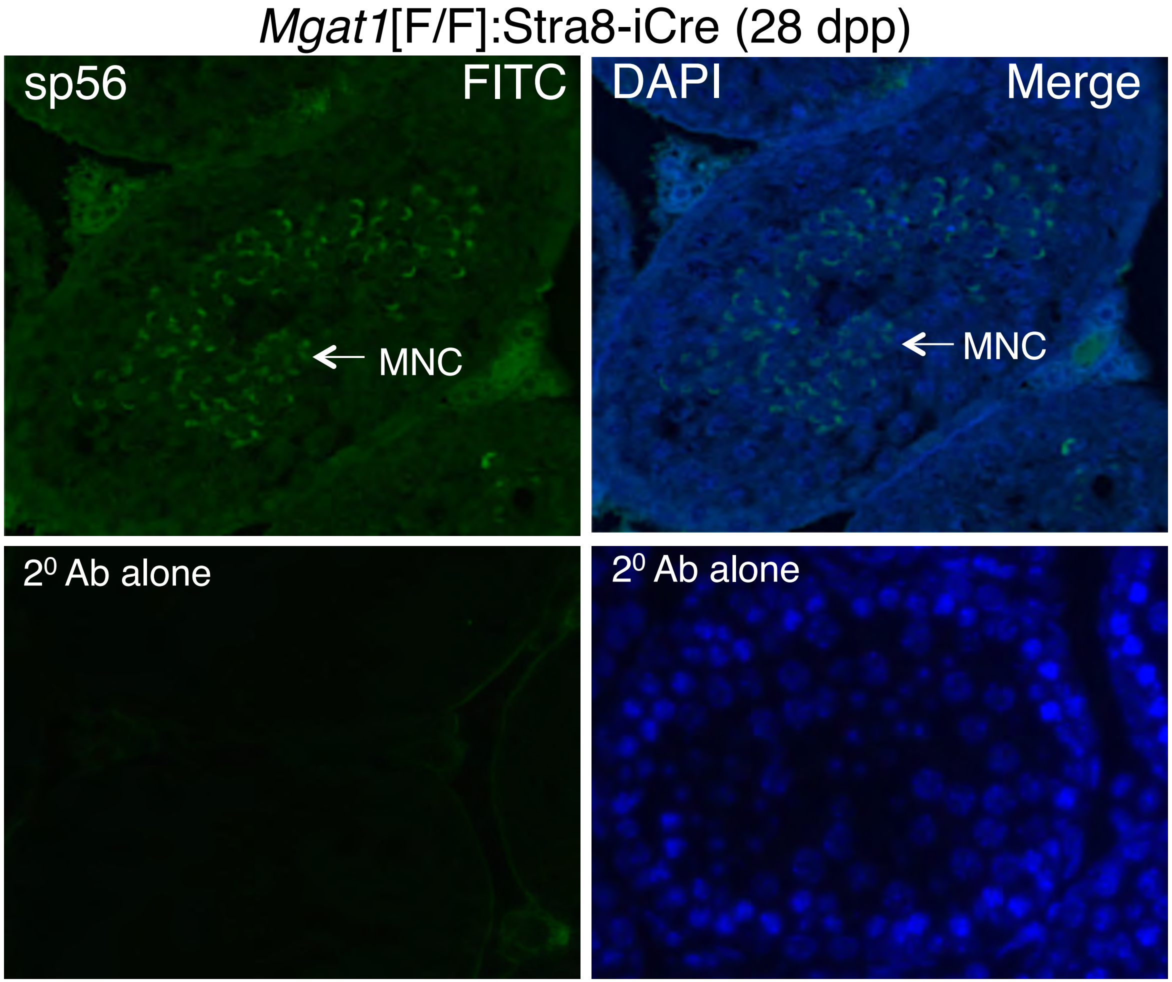

Mgat1[F/F]:Stra8-iCre (28 dpp)
sp56
FITC
DAPI
Merge
MNC
MNC
2⁰ Ab alone
2⁰ Ab alone

Figure S1

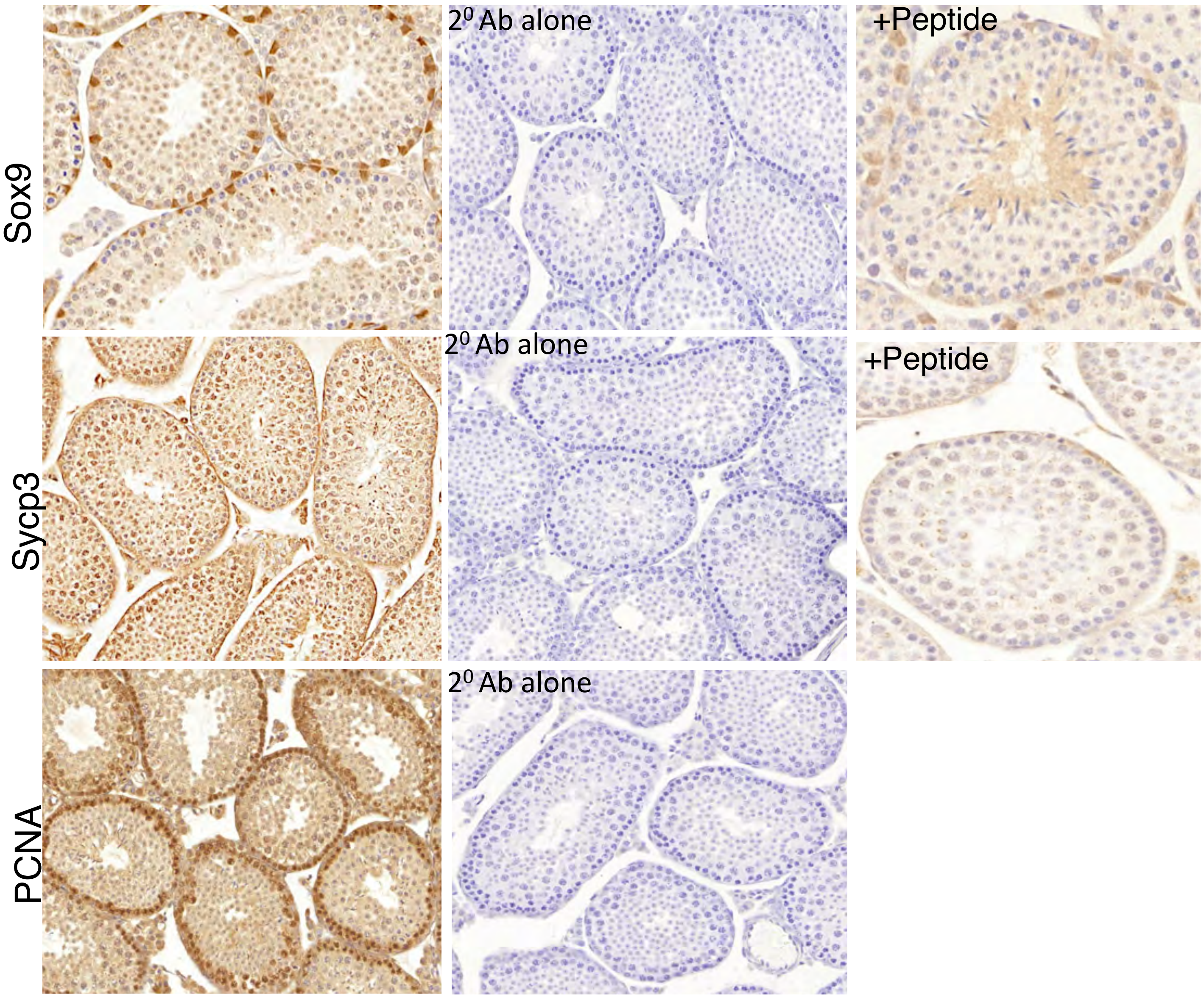
Sox9
Sycp3
PCNA
2⁰ Ab alone
2⁰ Ab alone
2⁰ Ab alone
+Peptide
+Peptide

Figure S2

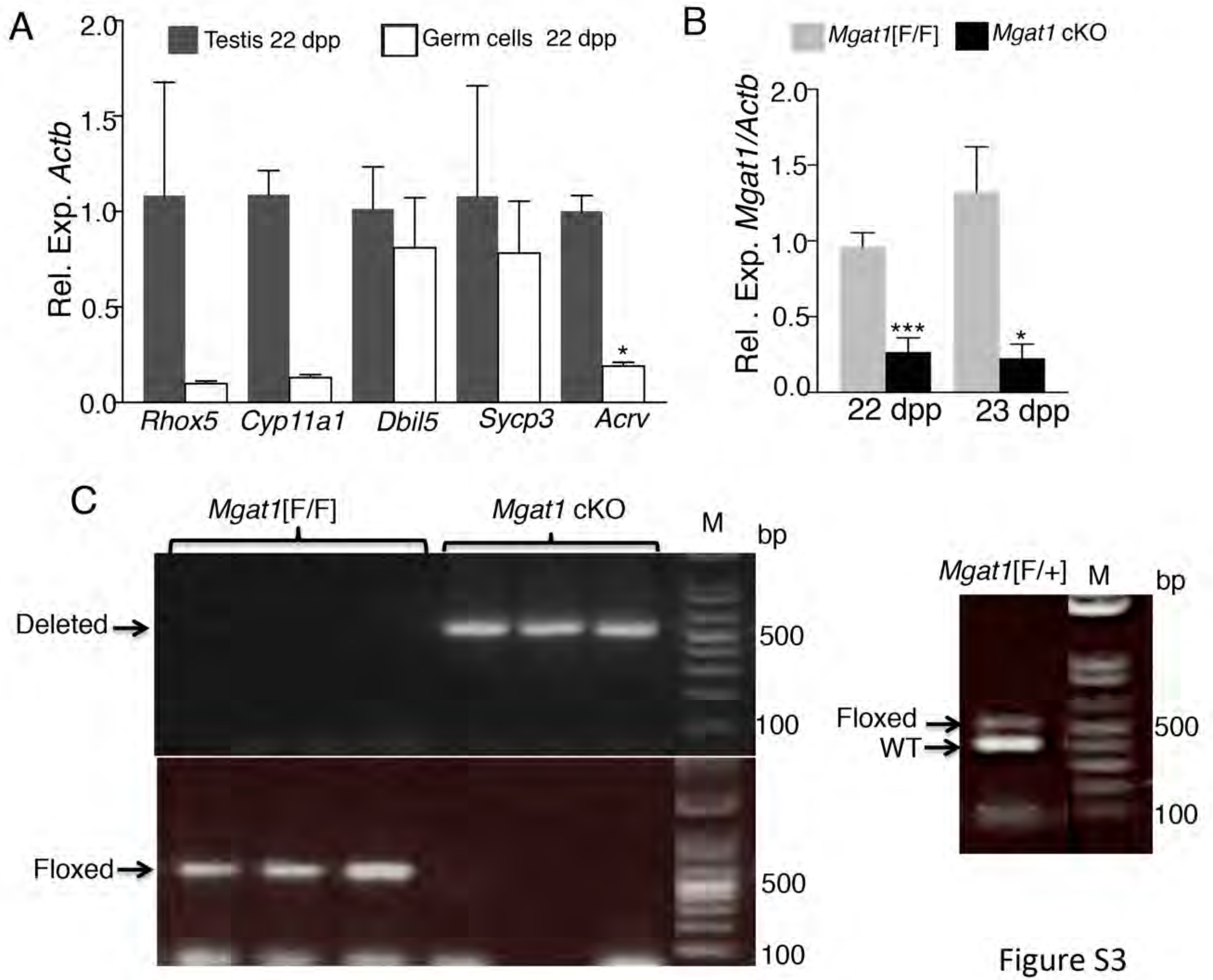
A
Testis 22 dpp
Germ cells 22 dpp
Rel. Exp. Actb
2.0
1.5
1.0
0.5
0.0
*
Rhox5
Cyp11a1
Dbil5
Sycp3
Acrv
B
Mgat1[F/F]
Mgat1 cKO
Rel . Exp. Mgat1/Actb
2.0
1.5
1.0
0.5
0.0
***
*
22 dpp
23 dpp
C
Mgat1[F/F]
Mgat1 cKO
M
bp
Deleted
500
100
Floxed
500
100
Mgat1[F/+]
M
bp
Floxed
WT
500
100

Figure S3

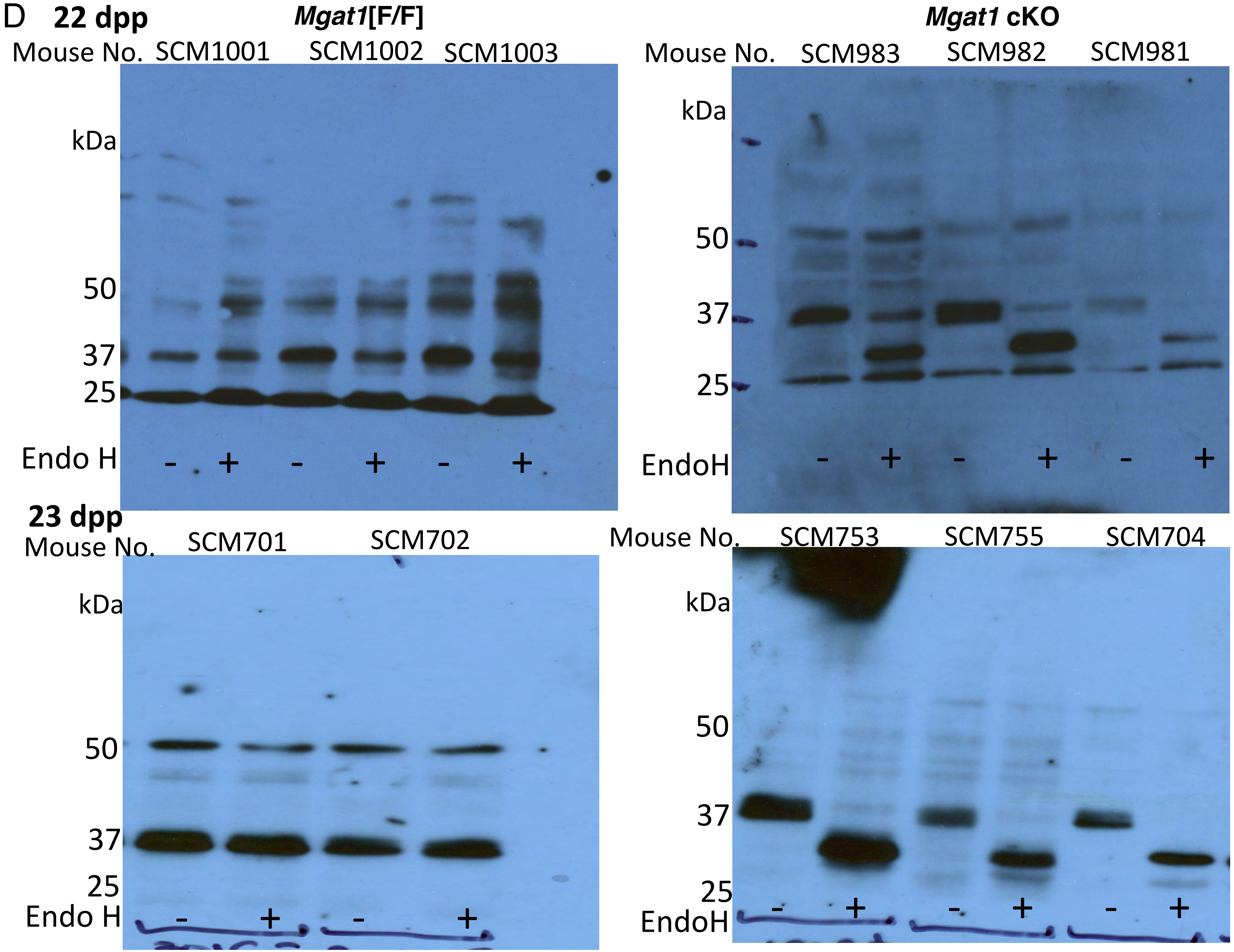
D
22 dpp
Mgat1[F/F]
Mouse No. SCM1001 SCM1002 SCM1003
kDa
50
37
25
Endo H - + - + - +
Mgat1 cKO
Mouse No. SCM983 SCM982 SCM981
kDa
50
37
25
EndoH - + - + - +
23 dpp
Mouse No. SCM701 SCM702
kDa
50
37
25
Endo H - + - +
Mouse No. SCM753 SCM755 SCM704
kDa
50
37
25
EndoH - + - + - +

Figure S3

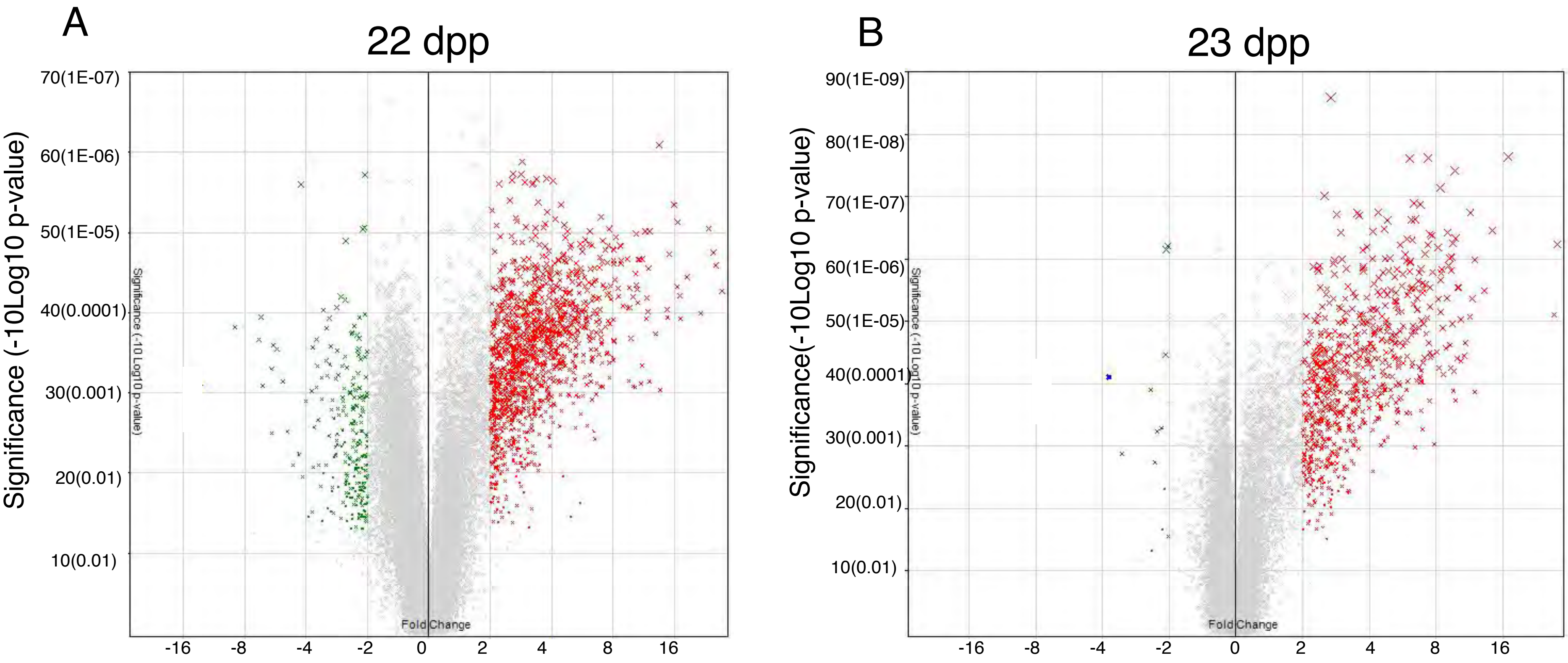
A
22 dpp
Significance (-10Log10 p-value)
70(1E-07)
60(1E-06)
50(1E-05)
40(0.0001)
30(0.001)
20(0.01)
10(0.01)
Fold Change
-16
-8
-4
-2
0
2
4
8
16
B
23 dpp
Significance(-10Log10 p-value)
90(1E-09)
80(1E-08)
70(1E-07)
60(1E-06)
50(1E-05)
40(0.0001)
30(0.001)
20(0.01)
10(0.01)
Fold Change
-16
-8
-4
-2
0
2
4
8
16

Figure S4

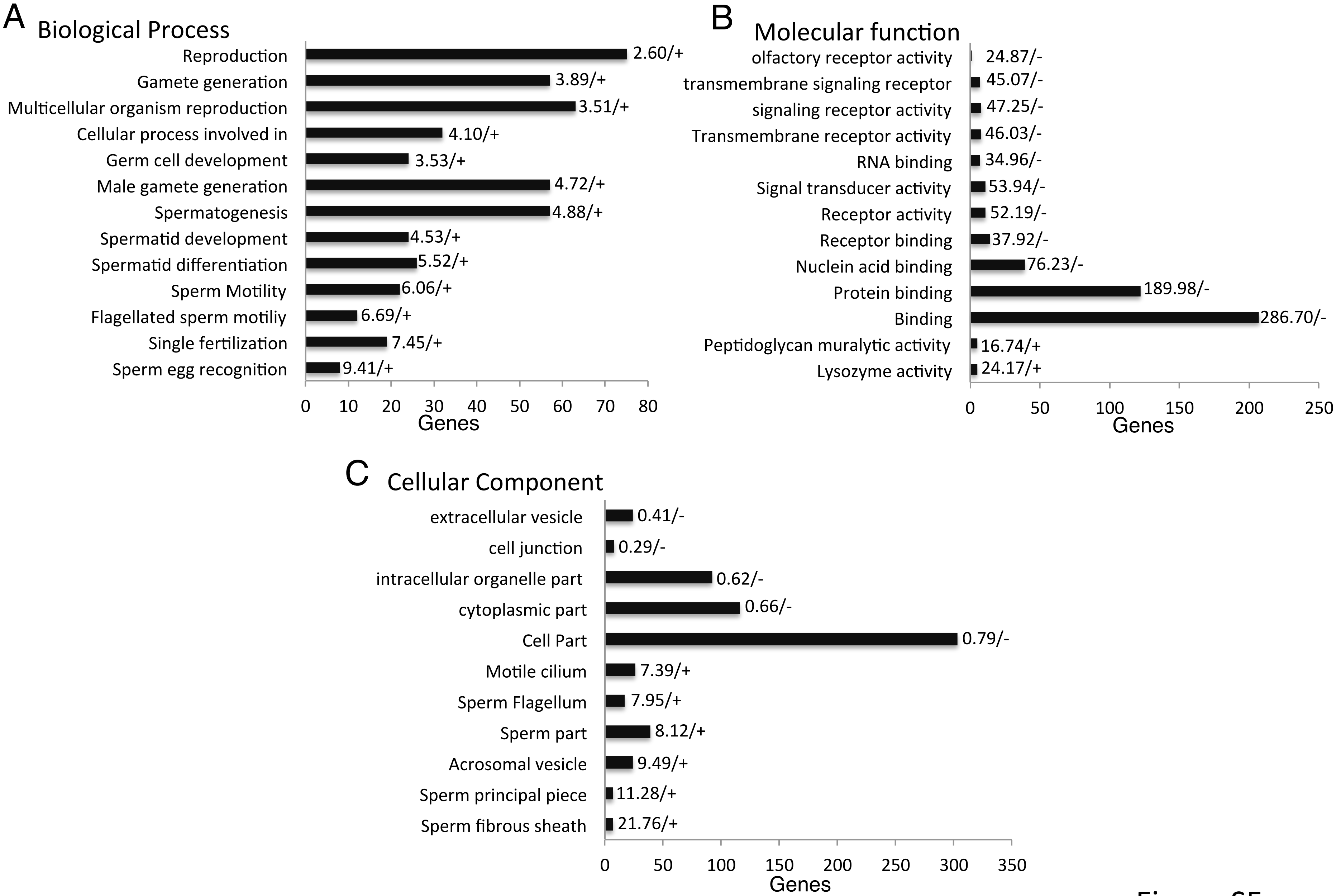

A
Biological Process
Reproduction 2.60/+
Gamete generation 3.89/+
Multicellular organism reproduction 3.51/+
Cellular process involved in 4.10/+
Germ cell development 3.53/+
Male gamete generation 4.72/+
Spermatogenesis 4.88/+
Spermatid development 4.53/+
Spermatid differentiation 5.52/+
Sperm Motility 6.06/+
Flagellated sperm motiliy 6.69/+
Single fertilization 7.45/+
Sperm egg recognition 9.41/+
0 10 20 30 40 50 60 70 80
Genes
B
Molecular function
olfactory receptor activity 24.87/-
transmembrane signaling receptor 45.07/-
signaling receptor activity 47.25/-
Transmembrane receptor activity 46.03/-
RNA binding 34.96/-
Signal transducer activity 53.94/-
Receptor activity 52.19/-
Receptor binding 37.92/-
Nuclein acid binding 76.23/-
Protein binding 189.98/-
Binding 286.70/-
Peptidoglycan muralytic activity 16.74/+
Lysozyme activity 24.17/+
0 50 100 150 200 250
Genes
C
Cellular Component
extracellular vesicle 0.41/-
cell junction 0.29/-
intracellular organelle part 0.62/-
cytoplasmic part 0.66/-
Cell Part 0.79/-
Motile cilium 7.39/+
Sperm Flagellum 7.95/+
Sperm part 8.12/+
Acrosomal vesicle 9.49/+
Sperm principal piece 11.28/+
Sperm fibrous sheath 21.76/+
0 50 100 150 200 250 300 350
Genes

Figure S5

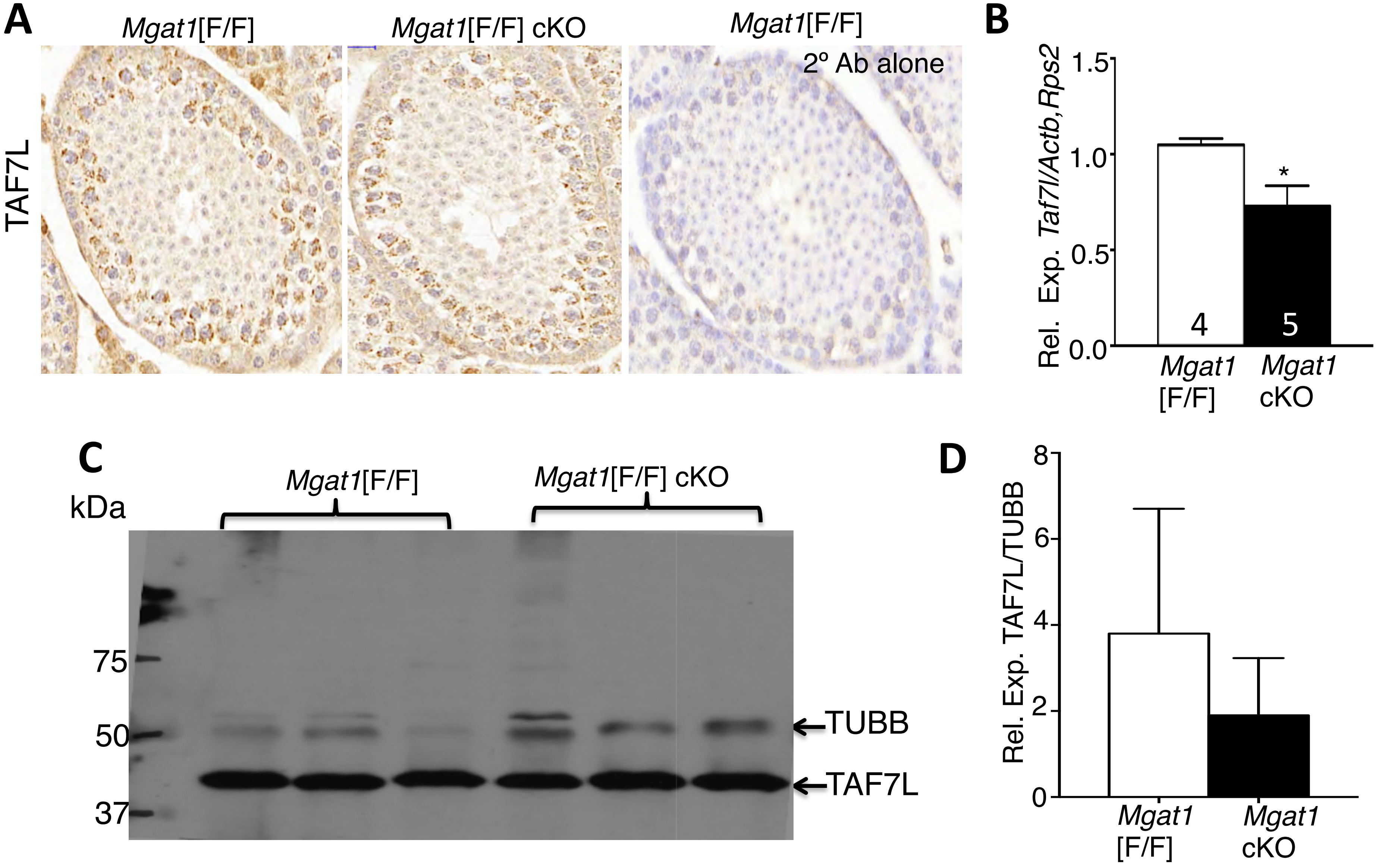
A
Mgat1[F/F]
Mgat1[F/F] cKO
Mgat1[F/F]
2º Ab alone
TAF7L
B
Rel. Exp. Taf7l/Actb,Rps2
1.5
1.0
0.5
0.0
*
4
5
Mgat1 [F/F]
Mgat1 cKO
C
Mgat1[F/F]
Mgat1[F/F] cKO
kDa
75
50
37
TUBB
TAF7L
D
Rel. Exp. TAF7L/TUBB
8
6
4
2
0
Mgat1 [F/F]
Mgat1 cKO

Figure S6

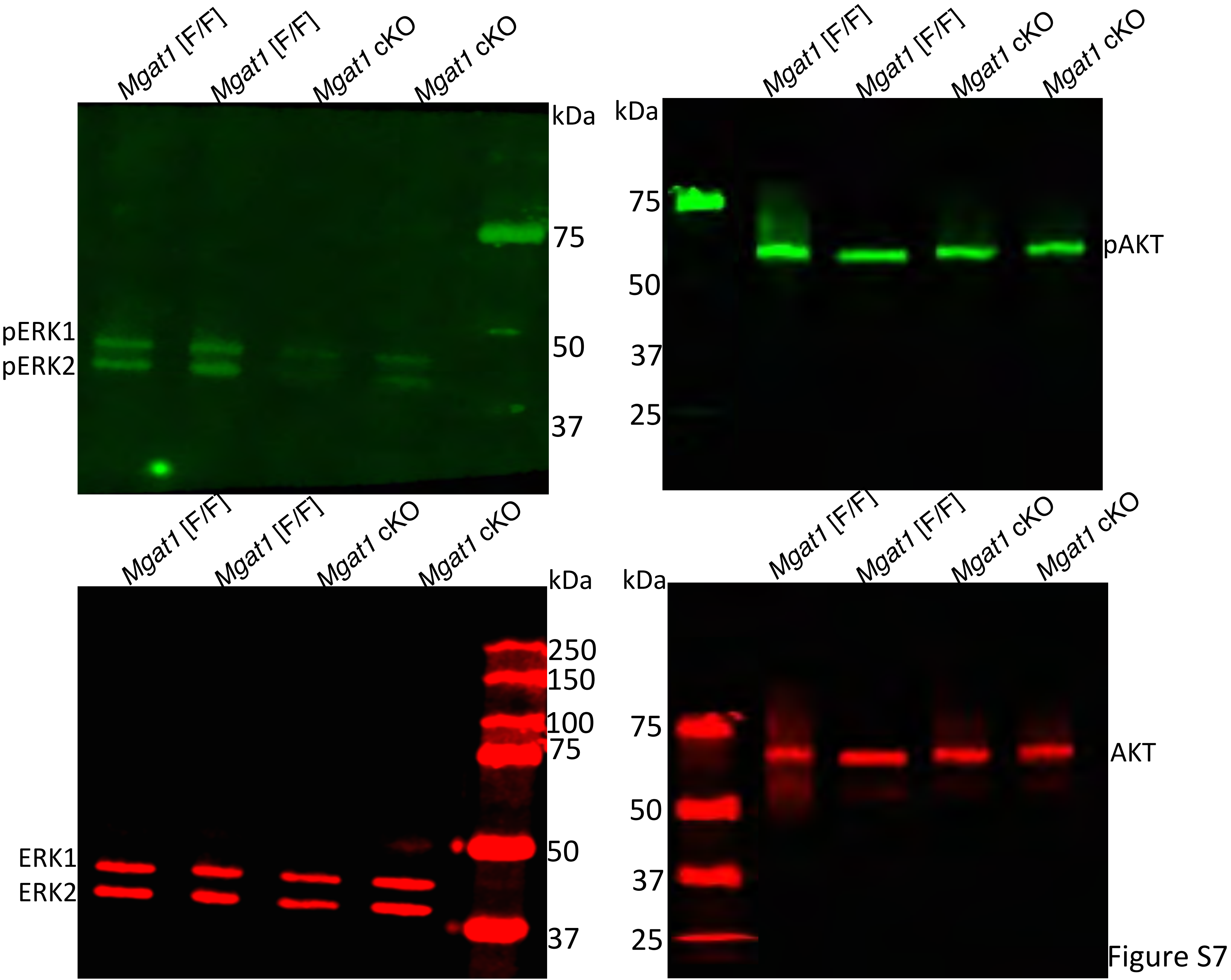
Mgat1 [F/F]
Mgat1 [F/F]
Mgat1 cKO
Mgat1 cKO
kDa
75
50
37
pERK1
pERK2
Mgat1 [F/F]
Mgat1 [F/F]
Mgat1 cKO
Mgat1 cKO
kDa
75
50
37
25
pAKT
Mgat1 [F/F]
Mgat1 [F/F]
Mgat1 cKO
Mgat1 cKO
kDa
250
150
100
75
50
37
ERK1
ERK2
Mgat1 [F/F]
Mgat1 [F/F]
Mgat1 cKO
Mgat1 cKO
kDa
75
50
37
25
AKT

Figure S7

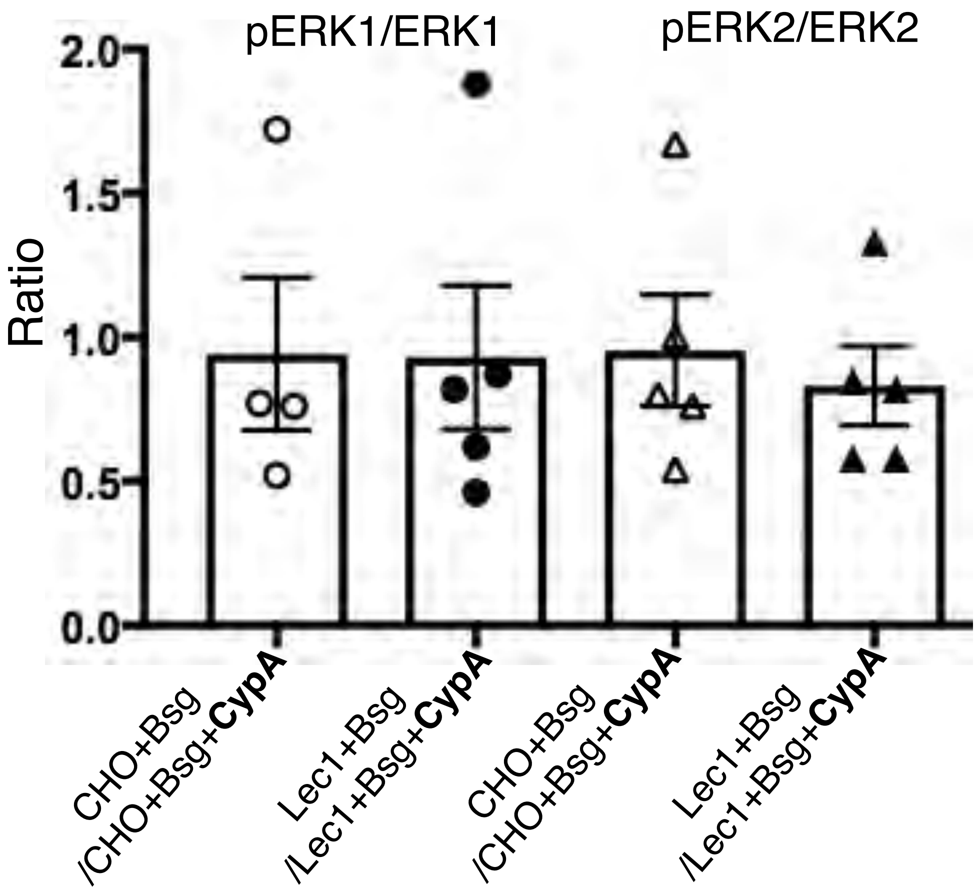
pERK1/ERK1
pERK2/ERK2
Ratio
2.0
1.5
1.0
0.5
0.0
CHO+Bsg
/CHO+Bsg+CypA
Lec1+Bsg
/Lec1+Bsg+CypA
CHO+Bsg
/CHO+Bsg+CypA
Lec1+Bsg
/Lec1+Bsg+CypA

Figure S8

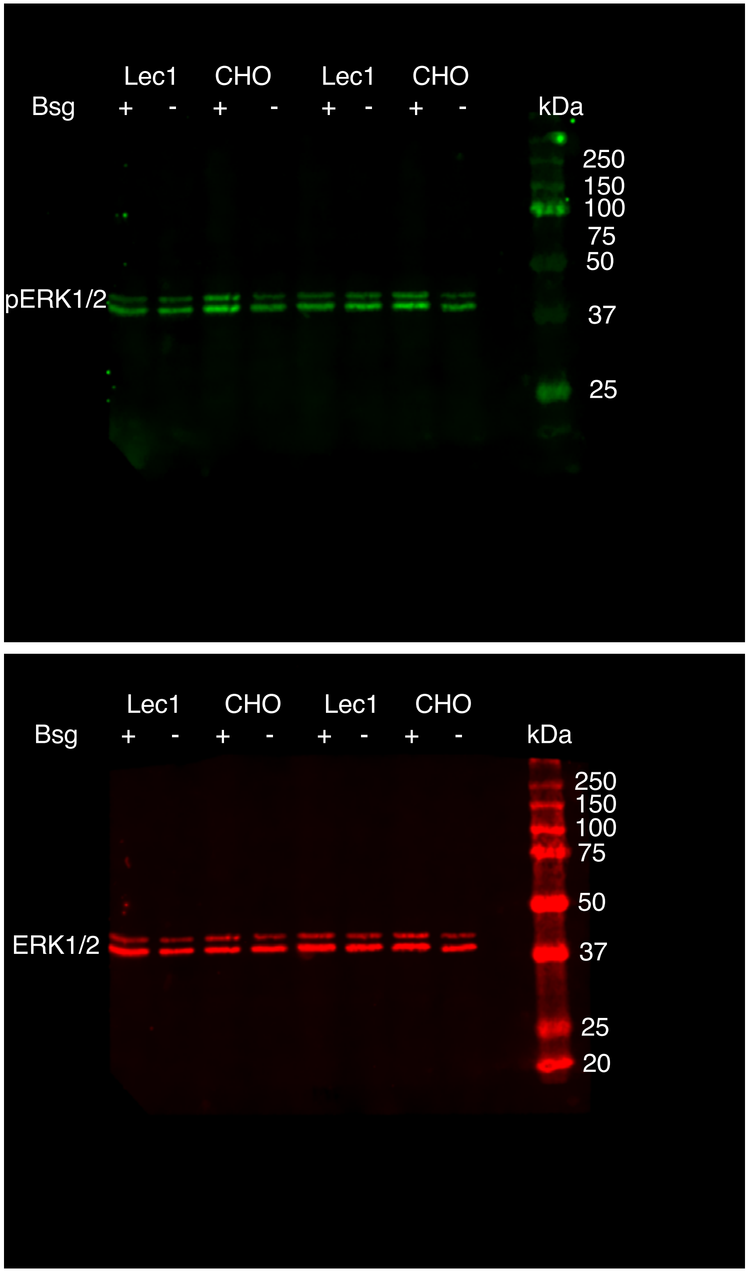

Lec1 CHO Lec1 CHO
Bsg + - + - + - + -
kDa
250
150
100
75
50
pERK1/2
37
25
Lec1 CHO Lec1 CHO
Bsg + - + - + - + -
kDa
250
150
100
75
50
ERK1/2
37
25
20

Figure S9
